# Lysosomal LRRC8 complex impacts lysosomal pH, morphology, and systemic glucose metabolism

**DOI:** 10.1126/sciadv.adt6366

**Published:** 2025-09-26

**Authors:** Ashutosh Kumar, Yonghui Zhao, Litao Xie, Rahul Chadda, John D. Tranter, Ryan T. Mikami, Nihil Abraham, Juan Hong, Ethan Feng, David R. Rawnsley, Haiyan Liu, Kayla M. Henry, Gretchen Meyer, Meiqin Hu, Haoxing Xu, Antentor Hinton, Chad E. Grueter, E. Dale Abel, Andrew W. Norris, Abhinav Diwan, Rajan Sah

**Affiliations:** ^1^ Department of Internal Medicine, Cardiovascular Division, Washington University School of Medicine, St. Louis, MO, USA.; ^2^ Fraternal Order of Eagles Diabetes Research Center, Iowa City, IA, USA.; ^3^ Division of Cardiology, Department of Internal Medicine, University of Iowa, Iowa City, IA, USA.; ^4^ Program in Physical Therapy and Departments of Neurology, Biomedical Engineering and Orthopedic Surgery, Washington University in St. Louis, St. Louis, MO, USA.; ^5^ Department of Molecular, Cellular, and Developmental Biology, University of Michigan, Ann Arbor, MI, USA.; ^6^ Department of Molecular Physiology and Biophysics, Vanderbilt University, Nashville, TN, USA.; ^7^ Department of Internal Medicine, Division of Endocrinology and Metabolism, Iowa City, IA, USA.; ^8^ Department of Medicine, David Geffen School of Medicine, University of California, Los Angeles, Los Angeles, CA, USA.; ^9^ Stead Family Department of Pediatrics, Endocrinology and Diabetes Division, Fraternal Order of Eagles Diabetes Research Center, University of Iowa, Iowa City, IA, USA.; ^10^ St. Louis VA Medical Center, St. Louis, MO, USA.

## Abstract

The lysosome integrates anabolic signaling and nutrient sensing to regulate intracellular growth pathways. The leucine-rich repeat–containing 8 (LRRC8) channel complex forms a lysosomal anion channel and regulates PI3K-AKT-mTOR signaling, skeletal muscle differentiation, growth, and systemic glucose metabolism. Here, we define the endogenous LRRC8 subunits localized to a subset of lysosomes in differentiated myotubes. We show that LRRC8A affects leucine-stimulated mTOR; lysosome size; number; pH; expression of lysosomal proteins LAMP2, P62, and LC3B; and lysosomal function. Mutating an LRRC8A lysosomal targeting dileucine motif sequence (LRRC8A-L706A;L707A) in myotubes recapitulates the abnormal AKT signaling and altered lysosomal morphology and pH observed in LRRC8A knockout cells. In vivo, LRRC8A-L706A;L707A knock-in mice exhibit increased adiposity, impaired glucose tolerance and insulin resistance associated with reduced skeletal muscle PI3K-AKT-mTOR signaling, glucose uptake, and impaired incorporation of glucose into glycogen. These data reveal a lysosomal LRRC8-mediated metabolic signaling function regulating lysosomal function, systemic glucose homeostasis, and insulin sensitivity.

## Teaser

Lysosomal LRRC8 channels regulate lysosomal pH, skeletal muscle signaling, insulin sensitivity, and systemic glycemia.

## INTRODUCTION

Lysosomes are single membrane-bound acidic organelles that participate in recycling extra- or intracellular macromolecules, regulate nutrient sensing, and serve as signaling hubs to maintain cellular homeostasis (*1*–*3*). Cytoplasmic cargo materials or membrane-bound receptor proteins are recycled by a regulated autophagic process. The endocytosed cargo material is initially sequestered within double membrane-bound autophagosome vesicles, which subsequently fuse with late endosomes or lysosomes to form autolysosomes. This fusion process results in the incorporation of various components, such as lysosomal membrane proteins, hydrolytic enzymes, ion channels, and transporters (*4*–*6*). Many ion channels and transporters exist within the lysosomal membrane, which regulates lysosomal membrane potential, pH, autophagy, cellular signaling, and systemic metabolism. For example, a loss-of-function mutation in TRPML (mucolipin 1), primarily a Ca^2+^ channel, found in late endosomes/lysosomes, results in dysfunction of lysosomal pH regulation (*7*). This leads to accumulated autophagosomes, compromised autophagy, and ultimately the development of lysosomal storage disorders (*8*–*10*). Similarly, gain-of-function mutations in CLC-7, a Cl^−^/H^+^ exchanger found within the lysosome, result in altered lysosome morphology and an increase in autophagosomes (*11*). Cells with this mutation exhibit compromised autophagy as they are unable to effectively degrade endocytosed cargo material and form enlarged endo-lysosome compartments (*11*). TMEM206, also known as the proton-activated chloride channel (PAC) (*12*) or acid-sensing osmolyte regulator (ASOR) (*13*), translocates from the plasma membrane to the endosome, maintaining an optimal endosomal pH by releasing Cl^−^ ions from the endosomal lumen (*12*). Ablation of the PAC concentrates intralysosomal Cl^−^, leading to hyperacidic endosomes (*12*). Furthermore, TMEM175 depletion induces lysosomal acidification, impaired protein degradation, and the aggregation of cellular cargo materials, suggesting the requirement of optimal pH regulation in late endosomes and lysosomes for preserved physiological function (*14*).

LRRC8A (leucine-rich repeat–containing protein 8A, also known as SWELL1) is an essential subunit of a heterohexameric LRRC8 channel complex, which consists of LRRC8A and other LRRC8 family proteins (LRRC8B/C/D/E). The LRRC8 complex contains a transmembrane pore domain and a C-terminal 15 to 17 Leucine Rich Repeat Domain (LRRD), and while it is predominantly considered a plasma membrane ion channel (*15*, *16*), it has recently been shown to be present in lysosomes and regulates cellular osmolarity (*17*). In addition, an unbiased genome-wide CRISPR screen revealed that LRRC8A-null cells exhibit enlarged dysfunctional lysosomes (*18*). We, and others, previously demonstrated the LRRC8 complex to be involved in the regulation of various signaling pathways, including insulin-PI3K-AKT2 signaling, adipocyte size, as well as skeletal muscle differentiation, AKT-mTOR and AMP-activated protein kinase (AMPK) signaling in vitro, and metabolism and systemic glucose homeostasis in vivo (*19*–*21*). In this study, we examine the contributions of the lysosomal LRRC8A channel complex in lysosome function, including AKT-mTOR signaling, nutrient sensing, autophagy, and systemic metabolism. We demonstrate that the LRRC8A complex is endogenously present in lysosomes and regulates leucine-mediated mTOR signaling, lysosomal morphology, pH, and function. Mutating LRRC8A C-terminal dileucine lysosomal targeting motifs to alanine in LRRC8A-L706A; L707A knock-in (LL:AA KI) mice is sufficient to reduce lysosomal localization while preserving plasma membrane translocation. LL:AA skeletal muscle cells recapitulate numerous features of LRRC8A knockout (KO) cells in terms of altered lysosomal morphology, pH, and expression of lysosomal markers. Moreover, LL:AA KI mice exhibit increased adiposity, impaired glucose, and insulin tolerance, as well as impaired insulin-stimulated PI3K-AKT2 and mTOR signaling, with euglycemic clamps revealing systemic insulin resistance and reduced glucose uptake in skeletal muscle.

## RESULTS

### LRRC8 complex resides in lysosomes in native tissues

**Fig. 1.**
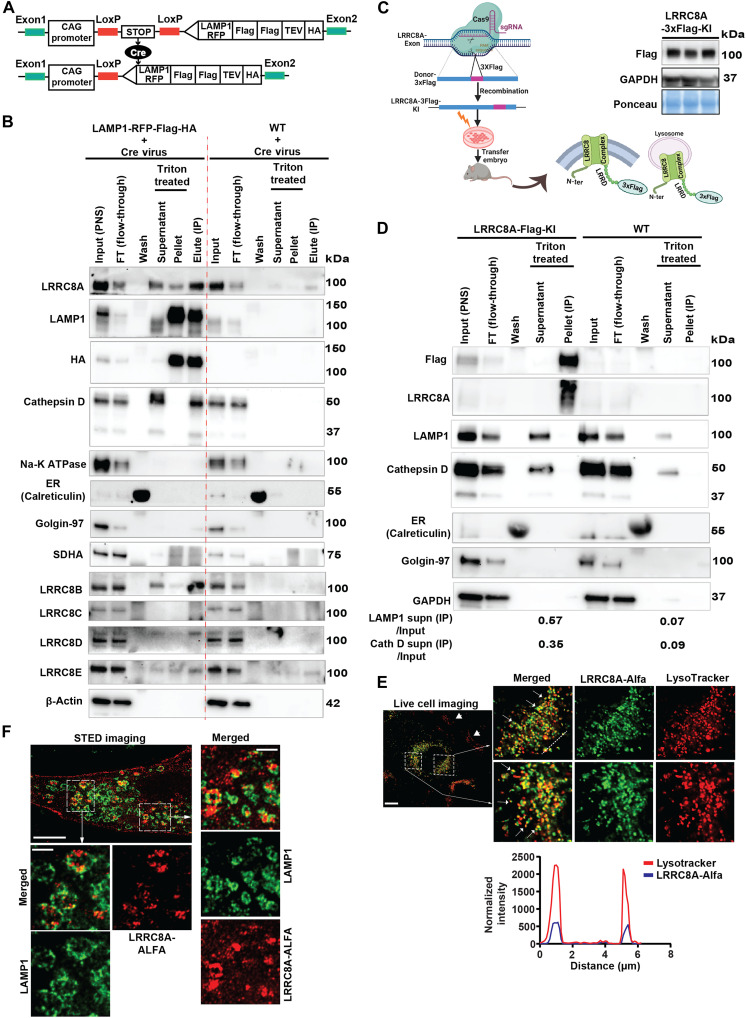
Endogenous LRRC8 proteins are present in lysosomes. (**A**) Schematic representation for the generation of Cre-inducible LAMP1-RFP-Flag-TEV-HA–expressing mouse. (**B**) Primary skeletal muscle myotubes isolated from Cre-inducible LAMP1-RFP-Flag-TEV-HA mice and C57BL/6 mice were transduced with Ad-CMV-Cre virus. Lyso-IP was performed with anti-HA magnetic beads and WB was performed for different LRRC8 complex subunits (LRRC8A/B/C/D/E); Input (postnuclear supernatant), FT (unbound protein), Wash (lysosome-bound beads washed with wash buffer), Triton X-100 treated (supernatant and pellet), and Elute (intact lysosomes bound with beads). WB of organelle-specific markers was performed to confirm the purity of isolated lysosome; Cathepsin D (lysosomal lumen protein), ER (Calreticulin), Golgi (Golgin-97), SDHA (Mito-ComplexII), Na-K ATPase (membrane marker), Lysosome (LAMP1), HA (epitope tag on expressed LAMP1), and Actin (loading control). (**C**) Schematic representation of the generation of 3×Flag-tagged LRRC8A knock-in (KI) mouse using the CRISPR-Cas9 approach. WB with anti-Flag antibody in isolated tibialis muscle of an LRRC8A-3×Flag (KI) mouse. GAPDH and Ponceau used as loading control (right side). (**D**) Lyso-IP performed on skeletal muscle tissue of LRRC8A-3×Flag-KI and WT control mice using anti-Flag magnetic beads. WB of Lyso-IP samples of LRRC8A-3×Flag (KI) in and WT for Flag, LRRC8A, LAMP1, Cathepsin D, ER, Golgi, and GAPDH protein. Densitometry quantification of LAMP1 and Cathepsin D shown below the images. (**E**) Live-cell confocal imaging showing colocalization of LysoTracker Red–stained lysosomes. White arrowheads indicate that nontransfected cells stain with LysoTracker Red only. Inset image shows that individual lysosome colocalizes with LRRC8A-ALFA. Scale bar, 10 μm. A line scanning intensity graph drawn between individual lysosome and LRRC8A-ALFA (lower side). (**F**) Stimulated emission depletion (STED) super-resolution imaging showing the localization of LRRC8A in the membrane and LAMP1-positive lysosomes (green). Inset images show an enlarged view of LRRC8A colocalization with lysosomes. Scale bars, 2 and 1 μm (inset). (C) was created in BioRender. Sah, R. (2025); https://BioRender.com/e33yijx.

Our previous work revealed that the adipocyte LRRC8 complex regulates PI3K-AKT2 signaling, adipocyte hypertrophy, and systemic glucose homeostasis (*19*). Similarly, skeletal muscle LRRC8 regulates AKT-mTOR, AMPK signaling, skeletal muscle size, and systemic glucose homeostasis in vivo (*20*). LRRC8-dependent mTOR signaling raised the possibility that LRRC8 may regulate lysosomal signaling (*20*). To explore this possibility biochemically in native tissue, we engineered a KI transgenic mouse that expresses epitope-tagged lysosome-associated membrane protein 1 (LAMP1; CAG-loxP-stop-loxP-LAMP1-RFP-2×Flag-TEV-HA) upon Cre-mediated excision of a floxed stop codon (Fig. 1A). Wild-type (WT) mice served as a negative control. We isolated and cultured primary skeletal muscle cells from these mice and then used adenoviral Cre to induce LAMP1-RFP-2×Flag-TEV-HA expression to allow for lysosomal immunoprecipitation (Lyso-IP). The eluted protein from LAMP1-RFP-Flag-HA–expressing cells (LAMP1-RFP-Flag-HA + Cre) reveals a strong LAMP1 and HA signal (Fig. 1B) with no LAMP1 or HA signal in WT negative control cells (WT + Cre). These LAMP1- and HA-positive lanes are negative for Calreticulin and Golgin-97 [Endoplasmic reticulum (ER) and Golgi markers, respectively], and show weak succinate dehydrogenase, subunit A (SDHA) signal (mitochondrial marker). The weak SDHA signal observed may be attributed to lysosome-associated mitochondria or mitophagy. Overall, these data are consistent with a relatively clean Lyso-IP, relatively free of other organelles. Furthermore, to confirm the membrane integrity of intact lysosomes bound with HA beads, we treated them with mild detergent (1% Triton X-100), collected supernatant and bead protein fraction, and immunoblotted for cathepsin D, a lysosomal lumen protein. The cathepsin D bands (precursor protein at 45 kDa and mature protein at 28 kDa) were observed only in the Triton X-100-treated supernatant fraction of LAMP1-RFP-Flag-HA–expressing cells, and not in the WT negative control lanes, suggesting that lysosomal membranes were intact during Lyso-IP (Fig. 1B). Moreover, the endogenous LAMP1 protein (lower band, without HA tag) released in the Triton X-100-treated supernatant is also consistent with intact lysosomal membranes during Lyso-IP. Furthermore, to rule out the possibility of plasma membrane contamination in the Lyso-IP lanes, we performed an immunoblot for Na-K ATPase (a plasma membrane marker). These data did not show any evidence of plasma membrane contamination in the Lyso-IP lanes as compared to input lanes (Fig. 1B). These LAMP1-HA+ organelles are positive for LRRC8A, in addition to LRRC8B, LRRC8D, and LRRC8E (Fig. 1B), while LRRC8C appears absent (Fig. 1B). The higher–molecular weight LRRC8D (upper band) observed in the Lyso-IP lanes has been previously reported in both muscle and kidney tissue (*21*, *22*) and has been interpreted as posttranslationally modified LRRC8D. Also, the LRRC8E band intensity in LAMP1-RFP-Flag-HA relative to the weak LRRC8E background signal in WT negative control cells implies lower lysosomal LRRC8E content. These data suggest that the lysosomal LRRC8 channel in skeletal muscle is primarily composed of LRRC8A, B, and D subunits, with trace amounts of LRRC8E subunits.

As a complementary biochemical approach, we generated LRRC8A-3×Flag KI mice in which the 3×Flag epitope was knocked into the endogenous locus of the LRRC8A C terminus after the leucine-rich repeat domain using CRISPR-Cas9 gene editing (Fig. 1C) to allow for endogenous immunoprecipitation (IP). On the basis of the topology of the LRRC8 channel complex, the C-terminal 3×Flag will be facing the cytoplasm, whether on the plasma membrane or within lysosomes (Fig. 1C) and therefore should be accessible for anti-Flag antibody–mediated Lyso-IP. Endogenous LRRC8A-3×Flag protein is detectable in mouse tibialis anterior (TA) using a Flag antibody (Fig. 1C, right). Next, using skeletal muscle tissue from LRRC8A-3×Flag KI mice as compared to WT controls with no 3×Flag KI, we isolated intact lysosomes and performed Lyso-IP using anti-Flag antibody (Fig. 1D). LRRC8A-3×Flag or LRRC8A protein is depleted in the flow through (FT; unbound protein) lane, indicating LRRC8A-3×Flag binding to the anti-Flag magnetic beads (Fig. 1D). Application of 1% Triton X-100 lysis buffer to the beads released LAMP1 and cathepsin D in the supernatant from LRRC8A-3×Flag–bound lysosomes and much less so from WT skeletal muscle, the latter reflecting a nonspecific binding of lysosome to the beads in WT samples (Fig. 1D). The LRRC8A-3×Flag Lyso-IP lanes are negative for Calreticulin (ER) and Golgin-87 (Golgi), consistent with containing lysosomes as opposed to other organelles. Densitometric quantification of Lyso-IP in Triton X-100-treated supernatant reveals an 8.1-fold LAMP1 enrichment and 3.9-fold cathepsin D enrichment relative to input in LRRC8A-3×Flag, as compared to WT controls (Fig. 1D). In addition to skeletal muscle, the Flag antibody–mediated Lyso-IP was performed in cardiac tissue of LRRC8A-3×Flag KI mice as compared to WT. The Triton X-100-treated beads of Lyso-IP samples of LRRC8A-3×Flag cardiac muscle release LAMP2 protein in the supernatant with minimum background in the WT lanes, suggesting the presence of intact endogenous lysosomes in the Lyso-IP lanes of LRRC8A-3×Flag KI mice as compared to WT (fig. S1). To get an enhanced immunoblot signal of endogenous LAMP2 from Flag-bound Lyso-IP, less protein was loaded in the FT lanes for both LRRC8A-3×Flag KI and WT control proteins than the input lanes. LRRC8A protein depletion is clearly observed in the LRRC8A-3×Flag KI FT as compared to WT control FT. Overall, this experiment provides a complementary approach to demonstrate lysosomal LRRC8 channels using Lyso-IP by pulling down endogenous LRRC8A-containing lysosomes from native skeletal and cardiac muscle tissues.

Next, as a complementary imaging approach, we generated an ALFA-epitope–tagged LRRC8A construct by placing an ALFA epitope tag within the first extracellular loop, yielding a versatile tool for both live-cell and fixed-cell imaging using high-affinity nanobodies (*23*) (fig. S2A). To confirm that ALFA-tagged LRRC8A forms a functional channel complex, we cotransfected LRRC8A-ALFA-IRES-EGFP and LRRC8C-P2A-mCherry in quintuple LRRC8A/B/C/D/E KO HeLa cells (5KO) and performed whole-cell patch-clamp recordings to measure hypotonically (210 mosmol) activated Volume-Regulated Anion Channel (VRAC) currents. LRRC8A-ALFA;LRRC8C channels are robustly activated by hypotonic swelling and completely inhibited by DCPIB (10 μM), a selective VRAC inhibitor (fig. S2, B to D), indicating that LRRC8A-ALFA forms a functional channel with LRRC8C. Next, we examined LRRC8A localization in live C2C12 myoblasts by transiently expressing LRRC8A-ALFA followed by confocal microscopy after pulse-chase application of anti–ALFA-Alexa 643–conjugated nanobody and LysoTracker staining (Fig. 1E). In transfected C2C12 myoblasts, ALFA-epitope–tagged LRRC8A colocalizes with LysoTracker-positive organelles, consistent with lysosomal LRRC8A. Furthermore, to rule out the possibility of nonselective endocytosis of ALFA antibody without epitope binding, we performed pulse-chase application of anti–ALFA-Alexa 643 antibody in untransfected LRRC8A KO C2C12 myoblast cells and performed confocal imaging. These results show negligible nonspecific endocytosis of ALFA antibody in these myoblast cells (fig. S3A). A line scanning intensity graph shows that LysoTracker-stained (red) puncta are also positive for LRRC8A protein (blue) (Fig. 1E, bottom). To further assess LRRC8A localization, we transfected LRRC8A-ALFA in LRRC8A KO C2C12 myoblast cells, performed immunostaining for LRRC8-ALFA and LAMP1 proteins in PFA-fixed cells, and imaged them using a stimulated emission depletion (STED) super-resolution microscope. STED imaging reveals LRRC8A to be present in the cell membrane and in intracellular puncta, forming a characteristic ring-like structure that colocalizes with LAMP1-positive lysosomal membranes (Fig. 1F and movie S1). STED imaging of untransfected LRRC8A KO C2C12 myoblast cells shows only LAMP1-positive lysosomes and an absence of ALFA-positive immunostaining (fig. S3B). Together, Lyso-IP using both LAMP1-RFP-2×Flag-TEV-HA–expressing mice and LRRC8A-3×Flag KI mice, as well as fluorescence imaging (live-cell confocal and fixed cell STED) in C2C12 myoblasts expressing ALFA-tagged LRRC8A, reveals LRRC8 to be present in a subset of lysosomes.

### LRRC8A regulates leucine-stimulated AKT-mTOR

**Fig. 2.**
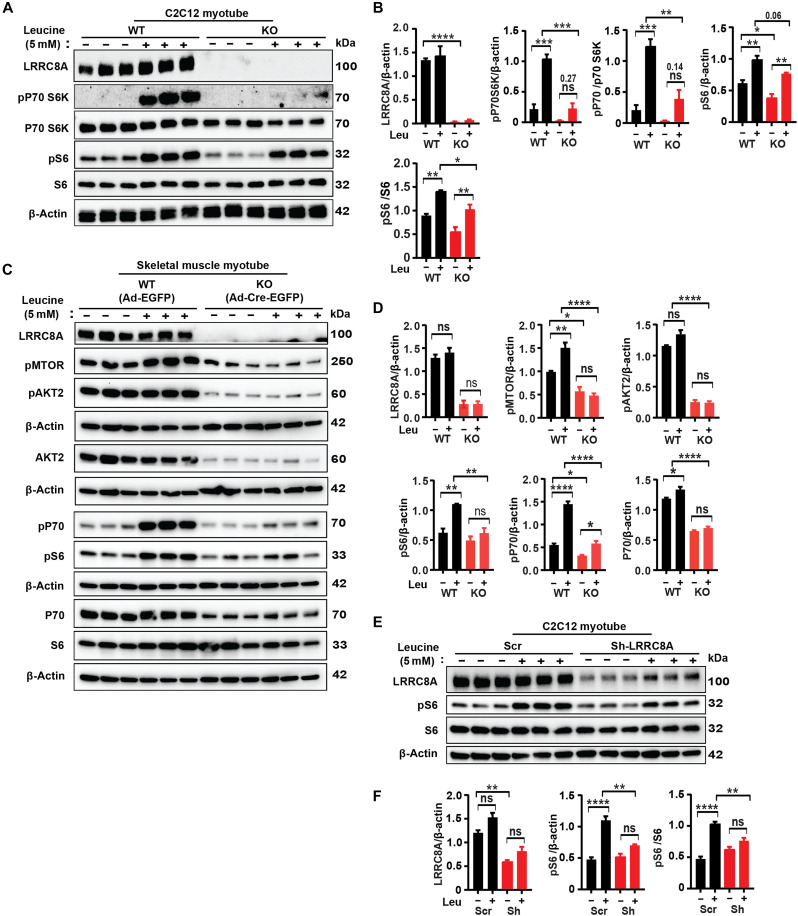
LRRC8A is required for leucine-stimulated mTOR signaling. (**A**) Western blot of LRRC8A, p-P70 S6k, P70 S6K, p-S6, S6, and β-actin in WT and LRRC8A KO C2C12 myotube after leucine (5 mM) stimulation for 15 min. (**B**) Densitometry quantification of leucine-stimulated signaling WB of (A). (**C**) WB of LRRC8A, β-actin, p-mTOR, p-AKT2, AKT2, p-P70 S6k, P70 S6K, p-S6, S6, protein in WT (LRRC8A^flfl^ + Ad-CMV-EGFP), and LRRC8A KO (LRRC8A^flfl^ + Ad-CMV-Cre-EGFP) primary myotube after leucine (5 mM) stimulation for 15 min. (**D**) Densitometry quantification of leucine-stimulated signaling WB of (C). (**E**) WB of LRRC8A, β-actin, and mTOR (pS6 and S6) signaling target protein in WT C2C12-sh-SCR and LRRC8A KD C2C12 myotube after leucine (5 mM) stimulation for 15 min. (**F**) Densitometry quantification of leucine-stimulated signaling WB (E). Statistical significance between the indicated values were calculated using statistical significance between the indicated group calculated with one-way ANOVA, Tukey’s multiple comparisons test. Error bars represent mean ± SEM. *P < 0.05, **P < 0.01, ***P < 0.001, ****P < 0.0001. ns, not significant. *n* = 3 independent experiments.

As the lysosome is central to amino acid nutrient sensing, we next examined leucine-stimulated AKT and mTOR signaling in LRRC8A KO skeletal myotubes. The branched-chain amino acid leucine and, to some extent, isoleucine are potent activators of mTOR signaling in various tissues (*24*). To assess for dose-dependent leucine-stimulated mTOR signaling, as reported earlier in muscle cells and other cell types (*25*, *26*), we stimulated C2C12 myotubes with three different concentrations of leucine (1, 2.5, and 5 mM) for 15 min. We observed a clear dose-dependent increase in downstream mTOR signaling, as assessed by p-S6 and p-P70 S6K, and all were consistently diminished upon LRRC8A depletion (fig. S4, A and B). On the basis of these studies, we stimulated C2C12 myotubes with 5 mM leucine to assess for mTOR signaling in subsequent experiments. Leucine (5 mM) stimulation induced robust mTOR signaling with phosphorylation of p70 S6K and S6, and this was markedly suppressed in LRRC8A-null cells (Fig. 2, A and B).

Stimulation of C2C12 myotubes with other amino acids, isoleucine and arginine, revealed a blunted pS6 and pP70 S6K response in both WT C2C12 and LRRC8A KO myotubes (fig. S4, C and D), as compared to leucine (Fig. 2, A and B). Arginine stimulation induced more robust pS6 in WT C2C12 as compared to LRRC8A KO myotubes, despite no notable change in the upstream mTOR protein pP70 S6K (fig. S4, E and F).

We next examined leucine-stimulated mTOR signaling in primary skeletal myotubes isolated and differentiated from LRRC8A^fl/fl^ mice and then treated with Ad-CMV-GFP or Ad-CMV-Cre-GFP to generate WT and LRRC8A-null primary skeletal myotubes, respectively (Fig. 2, C and D). Similar to C2C12 myotubes, WT primary skeletal myotubes exhibit clear increases in leucine-stimulated p-mTOR, p-P70, and p-S6 with no significant increases in leucine-stimulated pAKT2, and these were all diminished in LRRC8A-null myotubes (Fig. 2, C and D). As our previous work revealed that LRRC8A KO C2C12 cells exhibit impaired myotube differentiation (*20*), we determined whether the LRRC8A-dependent leucine-mediated downstream signaling defects in LRRC8A KO C2C12 cells are a result of impaired myotube differentiation, or whether this impairment is independent of the differentiation process. To test this, we first fully differentiated C2C12 cells and then knocked down (KD) LRRC8A protein by using Ad-shLRRC8A-mCherry followed by leucine stimulation (Fig. 2, E and F). Similar to LRRC8A KO C2C12 and primary skeletal myotubes, short-hairpin RNA (shRNA)–mediated LRRC8A KD in differentiated C2C12 myotubes reveal reductions in leucine-stimulated p-S6 protein (mTOR signaling) compared to WT control cells (Fig. 2, E and F), indicating that LRRC8A-dependent leucine signaling defects persist in differentiated myotubes.

### LRRC8A depletion alters lysosomal size, morphology, autophagic marker protein expression, and pH

**Fig. 3.**
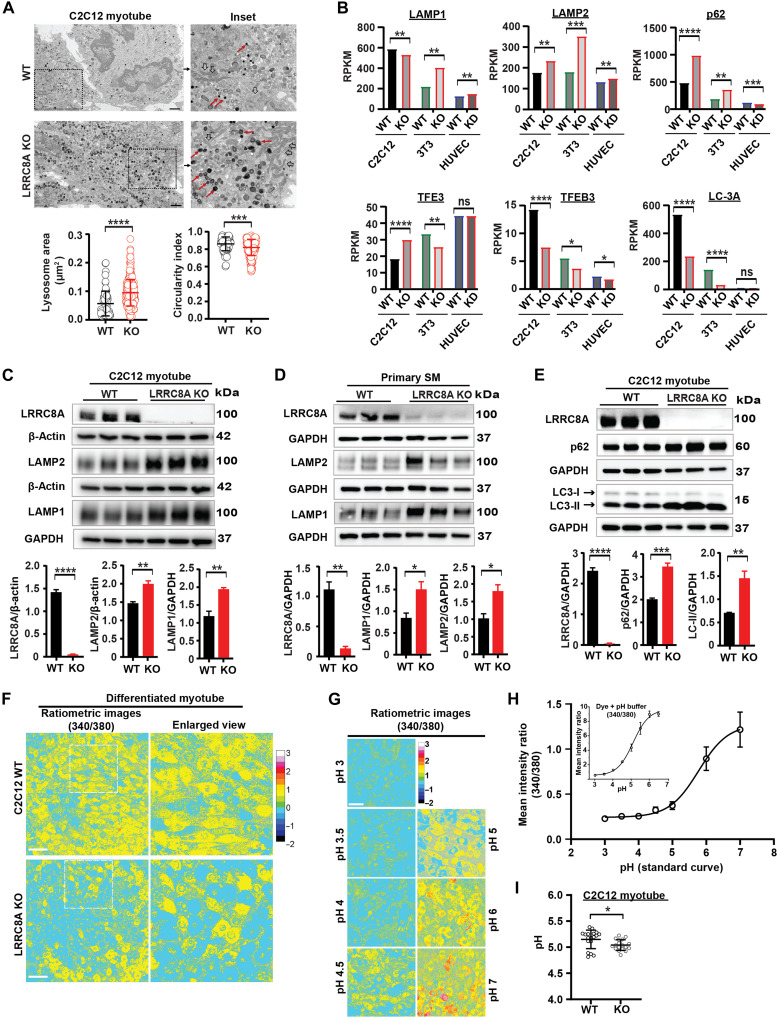
LRRC8A depletion increases lysosomal size, alters morphology and autophagic marker protein expression, and decreases pH. (**A**) Transmission electron microscopy (TEM) images of C2C12 WT and LRRC8A KO myotubes. Inset TEM image shows lysosome (red arrow) and mitochondria (black hollow arrow). Quantification of lysosome area and circularity index (WT = 73, LRRC8A KO = 265 lysosomes) (*n* = 2 independent experiments). Scale bar, 2 μm. (**B**) Reads per kilobase million (RPKM) for autophagy and lysosome biogenesis-related genes in C2C12 myotubes, 3T3-F442A adipocytes, and HUVECs. (**C** and **D**) WB of LRRC8A, LAMP1, LAMP2, β-actin, and GAPDH protein in WT and LRRC8A KO C2C12 myotube (C) and WT (LRRC8A^flfl^ + Ad-CMV-EGFP) and LRRC8A KO (LRRC8A^flfl^ + Ad-CMV-Cre-EGFP) primary myotubes, respectively (D); densitometry quantification below. (**E**) WB of LRRC8A, autophagy marker, and GAPDH protein in WT and LRRC8A KO C2C12 myotubes; densitometry quantification below. (**F**) Ratiometric images of Lysosensor-labeled WT and LRRC8A KO myotube. Scale bar, 100 μm. (**G**) Ratiometric images of WT C2C12 myotubes labeled with Lysosensor and incubated with different pH buffers. (**H**) pH standard curve plotted by using ratiometric image intensity at different pH buffers in WT C2C12 myotubes (*n* = 5, fluorescent images). Inset shows Lysosensor dye fluorescence at different pH buffers. (**I**) Lysosomal pH of WT and LRRC8A KO myotubes, which were determined from the nonlinear least squares fit to the pH calibration curve [total field of view 18 (WT) and 18 (KO), collected from six dishes per condition]. Statistical significance for (A), calculated by Mann-Whitney test. Error bars represent SD. [(B) to (E) and (I)] Significance calculated using a two-tailed Student’s *t* test. Error bars in (B) to (E) represent ±SEM, whereas those in (I) represent SD. One-way ANOVA used for (H), error bars represent SD. *P < 0.05, **P < 0.01, ***P < 0.001, ****P < 0.0001.

To more directly examine lysosomal morphology, we performed transmission electron microscopy (TEM) imaging of WT and LRRC8A KO C2C12 myotubes (Fig. 3A). Lysosomes, osmiophilic structures visualized under TEM, have a 67% larger surface area and 6% reduction in circularity index in LRRC8A KO C2C12 myotubes relative to WT myotubes (Fig. 3A). The lysosomal circularity index is closest to 1 when lysosomes are most compact, and it decreases when lysosomes become irregular or aggregated. To examine this cellular phenotype in a different cell type, we performed TEM imaging in human umbilical vein endothelial cells (HUVECs) treated with an adenoviral shRNA expressing a scrambled control (Ad-shSCR) or shLRRC8A (Ad-shLRRC8A), as described previously (*27*) (fig. S5). Upon shRNA-mediated LRRC8A KD, HUVECs also showed enlarged autophagosomes, increased lysosomal surface area, and decreased circularity index (fig. S5, A and B). Next, using RNA sequencing (RNA-seq) datasets from our previously published work (*19*, *20*, *27*), we compared RNA transcripts of lysosomal biogenesis and autophagy-associated genes in C2C12 myotubes, 3T3-F442A adipocytes, and HUVECs (Fig. 3B). These transcriptomic data from multiple different LRRC8A-depleted cell types reveal markedly altered gene expression of lysosomal biogenesis (*TFEB*, *TFEB3*, *LAMP1*, and *LAMP2*) and autophagy marker proteins (p62, LC-3A), implicating the LRRC8 channel complex as a regulator of lysosomal function and biogenesis. Aligned with transcriptomic data, LAMP1 and LAMP2 protein levels increase upon LRRC8A deletion in both C2C12 and primary skeletal myotubes (Fig. 3, C and D), consistent with enlarged lysosomes in LRRC8A KO C2C12 myotubes. As lysosomal proteins LAMP1 and LAMP2 regulate autophagosome fusion with lysosomes for ultimate lysosomal degradation, we investigated the autophagic marker proteins p62, LC3-I, and lipidated-LC3-II in WT and LRRC8A KO C2C12 myotubes (Fig. 3E). LRRC8A KO C2C12 myotubes show significantly increased p62 and LC3-II proteins, suggesting impaired autophagic flux despite having higher LAMP protein. Because maintenance of optimal lysosomal pH is an important regulator of cellular function and autophagic flux, we next measured lysosomal pH in WT and LRRC8A KO C2C12 myotubes (Fig. 3F) and myoblasts (fig. S6A) using the ratiometric pH (Ex 340/380) Lysosensor. After labeling with Lysosensor, WT C2C12 myotubes were exposed to buffers with pH values ranging from 3 to 7. A pH standard curve was obtained by analyzing the pH-sensitive changes in fluorescence intensity (Ex 340/380) from the acquired images (Fig. 3, G and H). Both LRRC8A KO C2C12 myotubes and myoblasts exhibit lower lysosomal pH compared to WT C2C12, as determined by the pH standard curve, consistent with increased lysosomal acidification (Fig. 3I and fig. S6B). As a control, treatment with the V-ATPase inhibitor bafilomycin A1 (Baf A1) increased pH and alkalinized lysosomes as expected (fig. S6B). Overall, these results suggest that lysosomal morphology and pH are regulated by the LRRC8 channel complex.

### Mutating the LRRC8A lysosomal targeting sequence (LRRC8A-L706A;L707A) selectively depletes LRRC8A in lysosomes

**Fig. 4.**
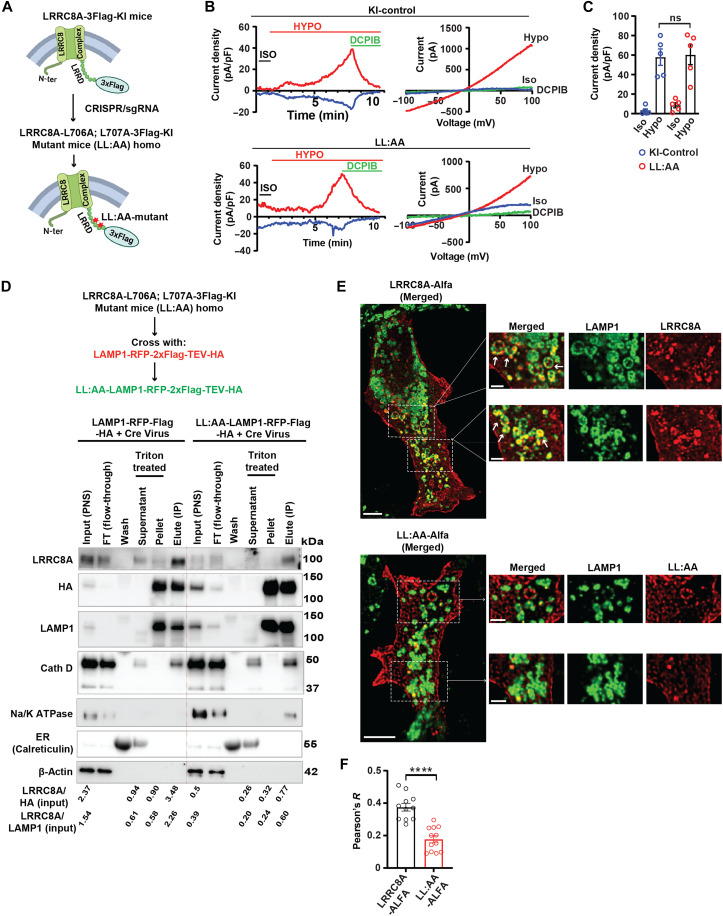
Lysosomal targeting sequence knock-in mutation LRRC8A-L706A;L707A selectively depletes LRRC8A in lysosomes in vivo. (**A**) CRISPR-Cas9–based approach to deplete lysosomal LRRC8 channels in vivo by introducing L706A;L707A double point mutations into the previously generated LRRC8A-3×Flag-KI mouse to generate LRRC8A-L706A;L707A-3×Flag KI (LL:AA) mice. (**B**) Whole-cell patch-clamp recordings of primary myoblasts isolated from LRRC8A-3×Flag (KI-control) and LRRC8A-L706A;L707A-3×Flag (LL:AA KI) showing current-time (left, at +100 and −100 mV) and current-voltage (right) relationships under isotonic (300 mosmol) and hypotonic (210 mosmol) conditions, followed by the application of 10 μM DCPIB. Voltage protocol applied was a voltage ramp −100 to +100 mV. (**C**) Mean outward current in isotonic and hypotonic conditions recorded from KI-control (*n* = 5) and LL:AA KI (*n* = 5) myoblasts. (**D**) Schematic representation for generating Cre-inducible LAMP1-RFP-Flag-TEV-HA;LRRC8A-L706A;L707A-3×Flag KI mice by crossing CAG-loxP-STOP-loxP-LAMP1-RFP-Flag-TEV-HA mice with LRRC8A-L706A;L707A-3×Flag KI mice. Primary skeletal muscle isolated from Cre-inducible LAMP1-RFP-Flag-TEV-HA mice and Cre-inducible LAMP1-RFP-Flag-TEV-HA;LRRC8A-L706A;L707A-3×Flag KI mice, transduced with Ad-CMV-Cre and lyso-IP performed with anti-HA beads. WB performed for LRRC8A, HA, Na/K ATPase, Cathepsin D, LAMP1, ER (Calreticulin), and β-actin. The ratios of LRRC8A/HA (input) or LRRC8A/LAMP1 (input) proteins in the Lyso-IP lane of WT-LAMP1 and LL:AA-LAMP1 are shown below. (**E**) Confocal imaging shows colocalization of LAMP1-positive lysosomes (green) with transiently expressed LRRC8A-ALFA (red) or LL:AA-ALFA in LRRC8A KO C2C12 myoblast cells. Inset image shows that individual lysosome colocalizes with LRRC8A-ALFA. Scale bars, 5 and 2 μm (inset). (**F**) Pearson’s relation for colocalization of LRRC8A-ALFA or LL:AA-ALFA with LAMP1 (LRRC8A-ALFA = 11, LL:AA-ALFA = 12, cropped area from five to six different cells). For (C) and (F), statistical tests for significance between the indicated values were carried out using a two-tailed Student’s *t* test. Error bars represent mean ± SEM. *P < 0.05, **P < 0.01, ***P < 0.001, ****P < 0.0001. (A) was created in BioRender. Sah, R. (2025); https://BioRender.com/b83qa0k.

Many lysosomal membrane proteins that contain lysosomal targeting dileucine motifs as a lysosomal targeting sequence are transported to lysosomes directly via an adaptor protein 2–dependent internalization process (*28*). The LRRC8A protein C terminus contains two consecutive dileucine motifs (LRRC8A-L706,L707), which are essential for lysosomal translocation. Mutation of these leucine residues to alanine (LRRC8A-L706A,L707A; LL:AA) is sufficient to abolish lysosomal localization while preserving plasma membrane translocation and function (*17*). To explore the physiological function of lysosomal LRRC8 channels, we engineered double point mutations in the *LRRC8A* gene on the background of LRRC8A-3×Flag KI (KI) mice to generate LRRC8A-L706A;L707A-3×Flag-KI (LL:AA) mice (Fig. 4A). To confirm whether LL:AA mutant channels still translocate to the plasma membrane and form functional channels, we isolated skeletal muscle myoblast cells from KI and LL:AA mice and performed whole-cell patch-clamp recordings to measure hypotonically (210 mosmol) activated VRAC currents. Hypotonic stimulation activated VRAC currents equally in both KI and LL:AA KI myoblasts, which were both completely inhibited by DCPIB (10 μM) (Fig. 4, B and C), indicating that LL:AA mutant forms a functional plasma membrane channel (Fig. 4, B and C).

Next, we generated LRRC8A-LL:AA;LAMP1-RFP-HA–tagged mice by crossing LRRC8A-LL:AA-3×Flag mice with transgenic LAMP1-RFP-HA mice (CAG-loxP-stop-loxP-LAMP1-RFP-2×Flag-TEV-HA) described earlier (Figs. 1A and 4A). We isolated primary skeletal myotubes from LAMP1-RFP-HA (WT) and LRRC8A-LL:AA-LAMP1-RFP-HA mice and then induced lysosomal HA-tagged LAMP protein expression by transducing with adenoviral-Cre, followed by Lyso-IP using HA-conjugated magnetic beads. The eluted and Triton X-100-treated protein from LAMP1-RFP-HA (WT) reveals the presence of LRRC8A in lysosomes (Fig. 4D) as previously observed (Fig. 1, B and D), with depletion of LRRC8A signal from LRRC8A-LL:AA-LAMP1-RFP-HA cells (Fig. 4D), indicating that introduction of the L706A; L707A dileucine mutation into LRRC8A-3×Flag mice selectively reduces lysosomal LRRC8 primary skeletal myotubes (Fig. 4D). LRRC8A-LL:AA-LAMP1-RFP-HA myotubes exhibit elevated levels of LAMP1-HA protein, with no changes in the levels of cathepsin D or actin proteins (input), consistent with our prior observations that LRRC8A depletion increases LAMP proteins levels (Fig. 3, C and D). Densitometry analysis of LRRC8A protein signal in Triton X-100-treated supernatant and eluted (IP) protein with respect to LAMP1-input (3.05- and 3.76-fold) or HA-input (3.61- and 4.51-fold) protein is enriched in LAMP1-RFP-HA (WT) lanes as compared to LAMP1-input or HA-input in LRRC8A-LL:AA mutant lanes (Fig. 4D). The eluted protein (IP) lane shows high LAMP1 and HA protein signals with almost no calreticulin (ER) protein signal, suggesting that HA-bead bound organelles are lysosomal with minimal contamination from the ER. To exclude the possibility of cell membrane protein contamination in the Lyso-IP lanes, we performed an immunoblot for the Na-K ATPase (plasma membrane marker) and observed no contamination of plasma membrane protein in the LAMP1-RFP-HA (WT) lanes, with minimal contamination in the LRRC8A-LL:AA mutant Lyso-IP lanes (Fig. 4D). This Na-K ATPase signal observed in the LL:AA-LAMP1-RFP-HA elute lane likely arises from the increased LAMP1-HA protein expression observed in the LL:AA-LAMP1-RFP-HA myotubes, which ultimately traffics via the plasma membrane to lysosomes. Na-K ATPase may then be immunoprecipitated with the plasma membrane pool of LAMP1-RFP-HA. This may also contribute to the “residual” LRRC8A protein observed in the Lyso-IP of LL:AA-LAMP1-RFP-HA myotubes. As a complimentary imaging approach, to visualize intracellular localization of WT LRRC8A-containing channels as compared to LRRC8A-LL:AA mutant channels, we performed confocal imaging of LRRC8A KO C2C12 myoblasts transfected with WT LRRC8A-ALFA versus LRRC8A-L706A,L707A-ALFA (LL:AA-ALFA). WT LRRC8A-ALFA translocates to the plasma membrane and colocalizes with a subpopulation of LAMP1-positive lysosomes (Fig. 4E), consistent with previous STED and live-cell imaging LRRC8A-ALFA results (Fig. 1, E and F). Notably, the LL:AA-ALFA mutant translocates to the plasma membrane but colocalizes poorly with lysosomes, consistent with previous work (*17*). Pearson’s correlation colocalization analysis reveals that the LL:AA-ALFA mutant exhibits significantly less colocalization with lysosomes compared to WT LRRC8A-ALFA (Fig. 4F).

### RNA-seq of lysosomal LRRC8A-depleted myotubes reveals differential expression of genes regulating inflammatory, autophagy, and metabolic signaling pathways

**Fig. 5.**
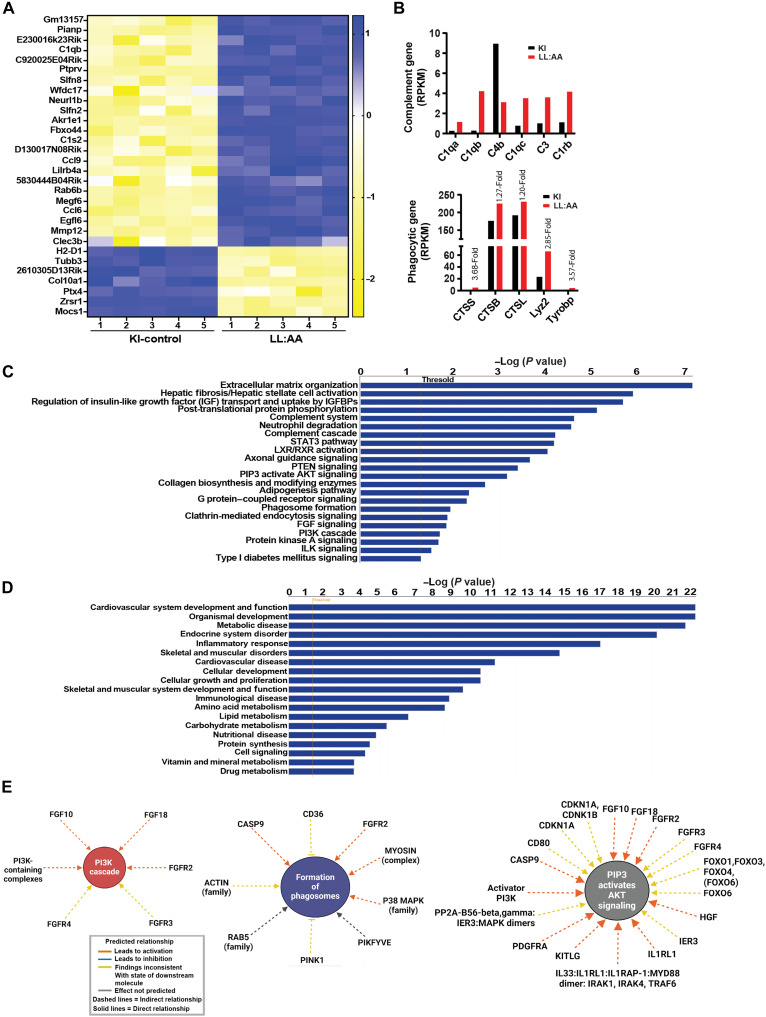
Bulk RNA transcriptome analysis of lysosomal LRRC8A-depleted myotubes reveals alterations in phagosome, inflammation, and metabolism-associated genes. (**A**) Heatmap analysis of the top 30 differentially expressed genes derived from RNA transcript of LRRC8A-3×Flag KI (*n* = 5) and LL:AA-3×Flag KI primary myotubes (*n* = 5). (**B**) Reads per kilobase million for selected gene of complement cascade, lysosomal cathepsin and phagocytic marker–associated genes. Fold change for phagocytic genes is displayed above the bar graph. (**C** and **D**) Ingenuity pathway analysis (IPA) of canonical pathways showing altered cellular signaling (C) and altered development, endocrine, and metabolic pathways (D) in LL:AA-3×Flag KI (*n* = 5) myotubes compared to LRRC8A3×Flag-KI (*n* = 5). For analysis with IPA, a fold change of ≥1.5 and a false discovery rate of <0.05 were used for significant differentially expressed genes. (**E**) Interaction networks identified by IPA shows affected cellular signaling pathways in LL:AA-3×Flag, including PI3K cascade (left), formation of phagosomes (center), and PIP3-activated AKT signaling (right). Network-associated gene names and line symbol are indicated by a predicted legend box (bottom).

To obtain an unbiased overview of the biological pathways affected by selective depletion of LRRC8 channels from lysosomes, we performed bulk RNA-seq in differentiated primary myotubes isolated from KI and LL:AA mice. Lysosomal LRRC8A depletion in primary myotubes enriched RNA transcripts of complement activation marker genes (*C1q*, *C1qb*, *C4b*, *C1qc*, *C3*, and *C1rb*) and lysosome-associated phagocytic genes such as cathepsin (*CTSS*, *CTSB*, and *CTSL*), lysozyme 2, and tyrosine motif binding protein (DAP12) (Fig. 5, A and B, and table S1). Hyperactivation of complement cascade-associated genes and dysfunctional cathepsin enzymatic activity serve as a hallmark of a dysfunctional lysosome-autophagy axis and are linked to lysosomal storage disorders (*29*–*33*). Furthermore, ingenuity pathway analysis (IPA) of RNA transcriptome data suggests that various essential cellular signaling pathways are down-regulated. These pathways include Phosphatase and Tensin Homolog (PTEN) signaling (4 × 10^−4^), Phosphatidylinositol-3,4,5-trisphosphate (PIP3) and AKT signaling (6 × 10^−4^), fibroblast growth factor signaling (1 × 10^−2^), PI3K signaling (1 × 10^−2^), and Integrin-linked kinase signaling (2 × 10^−2^) (Fig. 5C and table S2). In addition, inflammatory or phagocytic pathways such as Complement system (2 × 10^−5^), Complement cascade (6 × 10^−5^), neutrophil degranulation (2 × 10^−5^), STAT3 pathway (6 × 10^−5^), and phagosome formation (1 × 10^−2^) are also affected. These pathways are directly or indirectly associated with lysosome function (Fig. 5C and table S2). Moreover, the extracellular matrix (ECM) development pathway, which was the most highly activated canonical pathway by IPA, plays a crucial role in the adhesion of muscle fibers to the ECM. This pathway is an important regulator of muscle development and homeostasis and is regulated by lysosomal pH and the catabolic activity of lysosomes (*34*). In addition, the differentially expressed genes identified by the IPA-guided network analysis indicate various alterations in cellular signaling that are associated with metabolic disorders (Fig. 5, D and E, and fig. S7, A to D).

### Selective depletion of lysosomal LRRC8A recapitulates defects in lysosomal morphology, pH, and intracellular signaling observed in LRRC8A KO myotubes

**Fig. 6.**
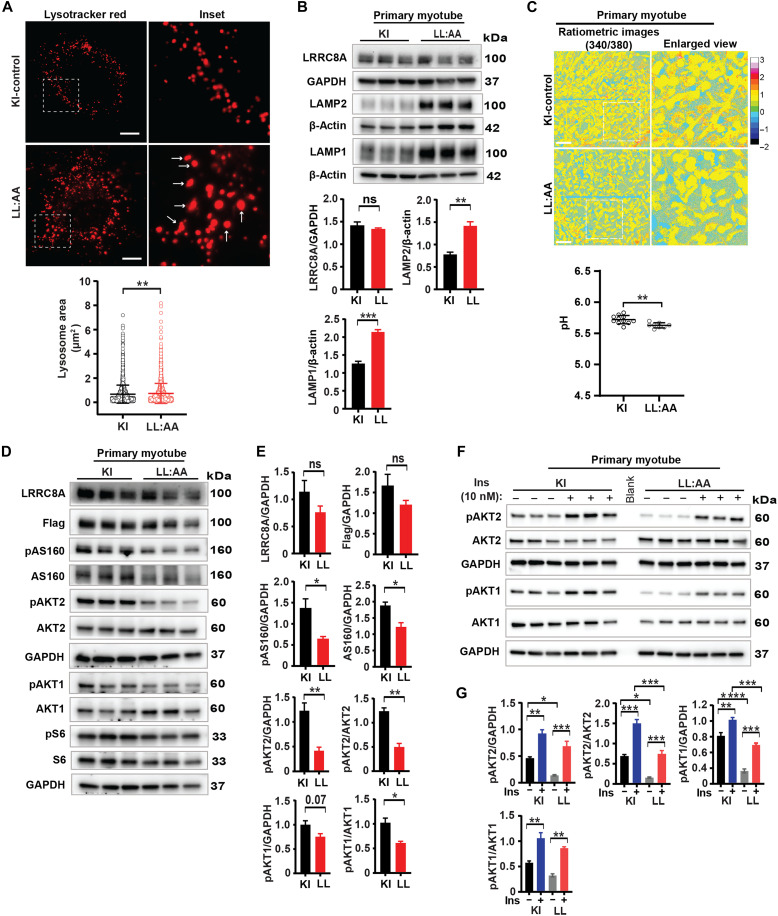
Lysosomal LRRC8A-depleted myotubes phenocopy LRRC8A KO myotubes with respect to lysosomal morphology, pH, and intracellular signaling. (**A**) Fluorescence image of LysoTracker Red–stained image of KI-control and lysosomal-depleted LRRC8A (LL:AA) primary muscle myotubes. Scale bar, 10 μm. Lysosome surface area quantified in KI-control (*n* = 2360 lysosomes) and LL:AA myotube (*n* = 1813 lysosomes) images. Error bars represent SD. (**B**) Western blot of LRRC8A, LAMP1, LAMP2, β-actin, and GAPDH protein in KI-control and LL:AA primary myotube. Densitometry quantification below. (**C**) Ratiometric (Ex340/Ex380) images of Lysosensor-stained images of KI-control and LL:AA primary myotubes. Scale bar, 100 μm. Lysosomal pH values of KI-control and LL:AA primary myotubes that were determined from the nonlinear least squares fit to the pH calibration curve [total field of view 11 (KI) and 8 (LL:AA), collected from six dishes per condition] (shown below). Error bars represent SD. (**D**) WB of LRRC8A, Flag, pAS160, AS160, pAKT2, AKT2, pAKT1, AKT1, and GAPDH protein in KI-control and LL:AA primary myotubes under basal conditions. (**E**) Densitometry quantification of (D). (**F**) WB of pAKT2, AKT2, pAKT1, AKT1, and GAPDH protein of KI-control and LL:AA primary myotubes stimulated with 0 and 10 nM insulin for 15 min. (**G**) Densitometry quantification of (F). Statistical significance for lysosome area (A) was calculated by Mann-Whitney test. For (B), (C), and (E), statistical tests for significance between the indicated values were carried out using a two-tailed Student’s *t* test. For (G), statistical significance between the indicated group was calculated with one-way ANOVA, Tukey’s multiple comparisons test. Error bars represent mean ± SEM. *P < 0.05, **P < 0.01, ***P < 0.001, ****P < 0.0001. *n* = 3 independent experiments.

Consistent with the pathway analysis of bulk RNA-seq data suggesting that lysosomal LRRC8 channels are important for phagosome function and biological processes that rely on phagocytic function (Fig. 5, A to E), LL:AA myotubes phenocopy LRRC8A KO/KD myotubes and LRRC8A KD HUVECs with respect to increases in lysosome size (Fig. 6A), induction of LAMP1/2 expression (Fig. 6B), and changes in lysosomal pH (Fig. 6C), with no significant change in total LRRC8A protein levels (Fig. 6B). These data indicate that specifically lysosomal LRRC8 localization/activity, rather than total LRRC8 activity, directly regulates lysosomal function, lysosomal homeostasis, and lysosomal pH.

Our previous work in skeletal muscle cells and adipocytes suggest that LRRC8A KO cells have impaired insulin-PI3K-AKT-mTOR signaling (*19*, *20*). To determine whether lysosomal LRRC8 channels contribute to insulin-PI3K-AKT-mTOR signaling, we isolated primary skeletal muscle cells from KI and LL:AA mice and examined the activity of these pathways under basal conditions (Fig. 6D). PI3K-AKT-pAS160 signaling is diminished in LL:AA skeletal myotubes under basal conditions, relative to KI cells, while mTOR signaling (pS6 and S6 ribosomal protein) is unchanged (Fig. 6, D and E). As a complementary approach, we reexpressed WT LRRC8A or mutant LRRC8A-LL:AA in LRRC8A KO C2C12 myotubes and examined basal PI3K-AKT-mTOR signaling. WT LRRC8A reexpression in LRRC8A KO C2C12 myotubes fully restored PI3K-AKT-mTOR signaling while LRRC8A-LL:AA reexpression failed to restore basal PI3K-AKT-mTOR signaling (fig. S8, A and B). We next examined insulin-stimulated PI3K-AKT2 signaling in KI and LL:AA primary myotubes. Consistent with the requirement of lysosomal LRRC8 channels for insulin-PI3K-AKT2 signaling, insulin-stimulated pAKT1 and pAKT2 are reduced in LL:AA myotubes compared to KI-control cells (Fig. 6, F and G). Overall, these data suggest that the lysosomal LRRC8 channel complex regulates lysosomal morphology, pH, and insulin-PI3K-AKT2 signaling in skeletal muscle cells.

### Lysosomal alkalinization with hydroxychloroquine and Baf A1 restores insulin signaling in LRRC8A KO myotubes

**Fig. 7.**
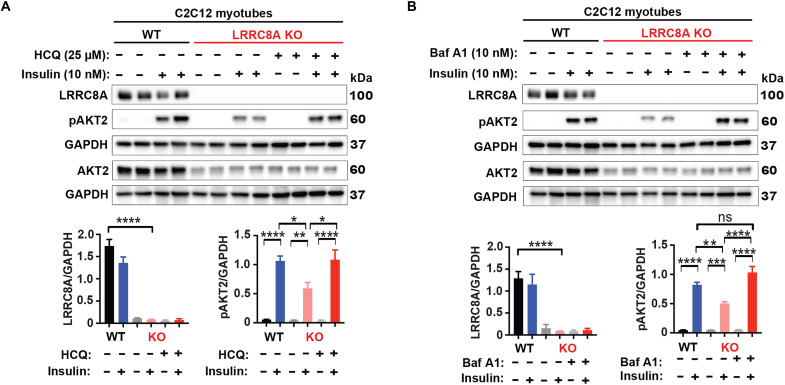
Lysosomal alkalinization restores insulin signaling in LRRC8A KO C2C12 myotubes. (**A** and **B**) Western blots of LRRC8A, pAKT2, AKT2, and GAPDH in LRRC8A KO myotubes, pretreated with either HCQ (25 μM) or Baf A1 (10 nM) for 4 hours and then stimulated with insulin (10 nM) for 15 min. Densitometry quantification of (A) and (B) WB shown below for each. Statistical significance between the indicated group calculated with one-way ANOVA, Tukey’s multiple comparisons test. Error bars represent mean ± SEM. *P < 0.05, **P < 0.01, ***P < 0.001, ****P < 0.0001. *n* = 3 independent experiments.

As lysosomal acidification is observed in both LRRC8A KO (Fig. 3, F and I) and lysosomal LRRC8A-depleted (Fig. 6C) myotubes, we hypothesized that decreased lysosomal pH could be responsible for impaired LRRC8A-dependent insulin-PI3K-AKT2 signaling by accelerating the termination of insulin signaling (*35*, *36*) as opposed to general lysosomal dysfunction and nonspecific cellular toxicity associated with LRRC8 channel depletion. To test this, we performed a subset of rescue experiments in LRRC8A KO myotubes by alkalinizing the lysosomes with either hydroxychloroquine (HCQ) or V-ATPase inhibitor Baf A1 for 4 hours, followed by insulin stimulation. Immunoblot results show that the impaired insulin-stimulated pAKT2 observed in LRRC8A KO C2C12 myotubes relative to WT myotubes is fully restored upon lysosomal alkalinization with either HCQ or Baf A1 (Fig. 7, A and B), and measurements of lysosomal pH in myotubes treated with HCQ or Baf A1 confirmed lysosomal alkalization (fig. S9, A and B). Mechanistically, HCQ is thought to enhance insulin signaling (*37*) and exhibit hypoglycemic activity in vivo (*38*, *39*), slowing down the dissociation and degradation of endosomal IR with insulin (*40*, *41*).

### LRRC8A-LL:AA KI mice exhibit impaired glucose tolerance, insulin resistance, and increased adiposity

**Fig. 8.**
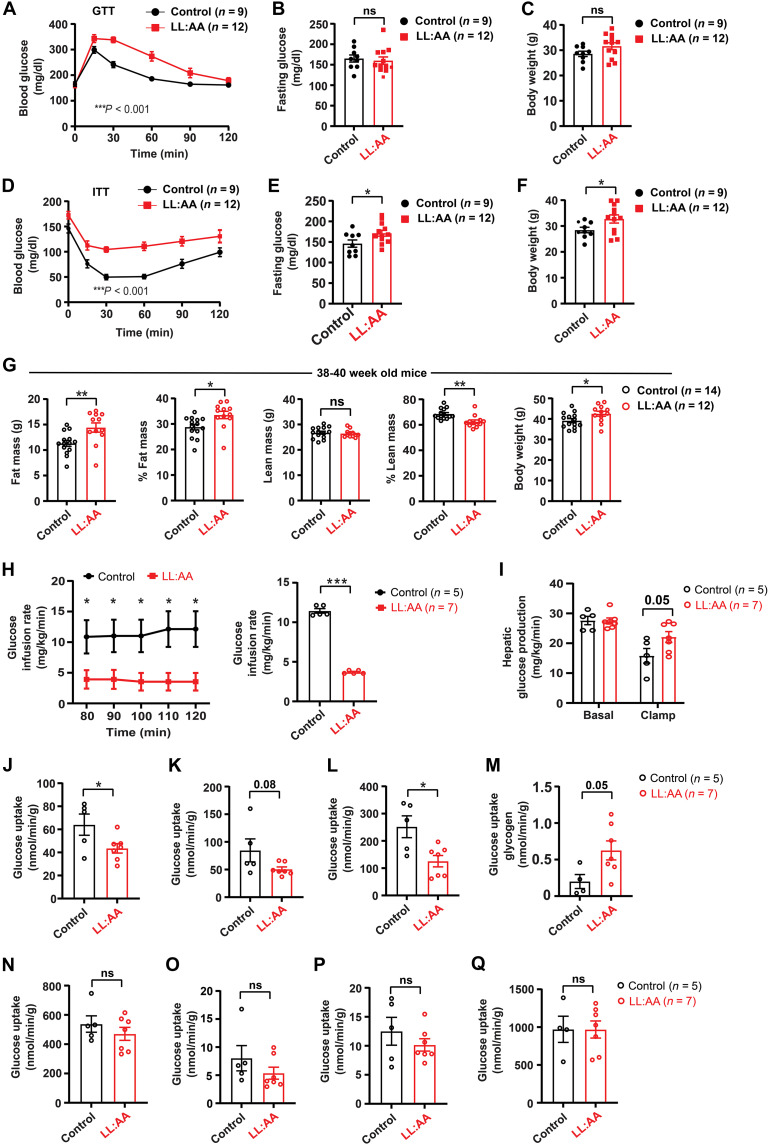
Lysosomal LRRC8A-depleted mice exhibit increased adiposity, impaired glucose tolerance and insulin resistance, and decreased glucose uptake. (**A**) Glucose tolerance test (GTT) of KI-control (*n* = 9) and LL:AA KI (*n* = 12) mice raised on chow diet for 20 to 22 weeks. (**B**) Six-hour fasting glucose during GTT. (**C**) Body weight. (**D** to **F**) Insulin tolerance test (ITT) of KI-control (*n* = 9) and LL:AA KI (*n* = 12) mice raised on chow diet for 22 to 24 weeks (D), fasting glucose after 4 hours (E), and body weight (F). (**G**) NMR measurement of absolute fat mass, % fat mass, absolute lean mass, % lean mass, and body weight of KI-control (*n* = 14) and LL:AA KI (*n* = 12) mice raised on chow diet for 38 to 40 weeks. (**H**) Average glucose-infusion rate during the euglycemic-hyperinsulinemic clamp period of KI-control (*n* = 5) and LL:AA KI (*n* = 7) mice on chow diet for 32 to 36 weeks, and mean glucose-infusion rate during the entire clamp period (70 to 120 min) on the right. (**I**) Hepatic glucose production at baseline and during the euglycemic-hyperinsulinemic clamp period. (**J** to **Q**) Glucose uptake determined from radiolabeled 2-deoxyglucose (2-DG) uptake in gastrocnemius (J), tibialis (K), soleus (L) muscle, liver (M), heart (N), inguinal white adipose tissue [iWAT, (O)], gonadal white adipose tissue [gWAT, (P)], and brown adipose tissue [BAT, (Q)] during the traced clamp period. Data were presented as mean ± SEM. Statistical test two-way ANOVA was used for (A), (D), and (H) (*P* value in the bottom corner of the graph). Error bars represent mean ± SEM. Two-tailed Student’s *t* test were done for all other data. *P < 0.05, **P < 0.01, ***P < 0.001, ****P < 0.0001.

In vitro experiments using LRRC8A KO C2C12 myotubes reexpressing LRRC8A-LL:AA and primary myotubes isolated from LRRC8A-LL:AA mice suggest that lysosomal LRRC8 regulates lysosomal function, autophagy, and insulin-PI3K-AKT2 signaling. To examine the physiological consequences of lysosomal LRRC8 depletion on systemic metabolism, we performed glucose tolerance tests (GTTs) in KI and LL:AA mice raised on regular chow diet for 20 to 22 weeks. Glucose tolerance was significantly impaired (Fig. 8A) in LL:AA mice relative to KI, with no significant difference in 6-hour fasting glucose (Fig. 8B), and a trend toward increased body weight (Fig. 8C). This glucose intolerance was associated with impaired insulin sensitivity (Fig. 8D) at 22 to 24 weeks of age, increased fasting glucose after a 4-hour fast, and a mild, but statistically significant increase in body weight (Fig. 8, E and F), suggesting LL:AA mice are more prone to obesity than KI mice over time. Consistent with this notion, LL:AA mice have increased body weight at 38 to 40 weeks of age (Fig. 8, E and F), and body composition analysis reveals this to be due to increased adiposity, with increased fat mass, increased % fat mass, and no change in lean mass (Fig. 8G). These data indicate that lysosomal LRRC8A depletion induces insulin resistance and increased adiposity with time in mice raised on a regular chow diet.

To further evaluate insulin sensitivity and determine the fate of glucose in metabolically important tissues that regulate insulin sensitivity, we performed euglycemic-hyperinsulinemic clamps traced with ^3^H-glucose and ^14^C-deoxyglucose (2-DG) in in LL:AA and KI-control mice raised on chow diet for 32 to 36 weeks. Clamp results indicate that LL:AA mice require a 68% lower glucose-infusion rate (GIR) to maintain euglycemia than KI mice, indicating reduced systemic insulin sensitivity (Fig. 8H). The rate of glucose appearance (R_a_), a measure of hepatic glucose production via gluconeogenesis or glycogenolysis, is increased 40% in LL:AA mice relative to KI mice during hyperinsulinemia, with no change at the basal level, consistent with impaired suppression of hepatic gluconeogenesis and hepatic insulin resistance (Fig. 8I). Next, we measured glucose uptake in individual tissues using 2-DG. Glucose uptake is reduced in a number of skeletal muscle tissues in LL:AA mice relative to KI mice: 32% in gastrocnemius, 50% in soleus, and a notable reduction of 40% in TA muscle groups, with no significant differences in glucose uptake in heart, adipose tissue (gonadal white adipose tissue and inguinal white adipose tissue), and brown adipose tissue (Fig. 8, J to Q). Incorporation of glucose into hepatic glycogen is higher in the LRRC8A-LL:AA-KI mice during the clamp period (Fig. 8M), despite an increase in rate of glucose appearance secondary to increased gluconeogenesis/glycogenolysis (Fig. 8I). Overall, these experiments reveal that LL:AA mice exhibit systemic insulin resistance primarily driven by impaired insulin sensitivity of skeletal muscle, and possibly liver, with compensatory increases in adiposity over time.

**Fig. 9.**
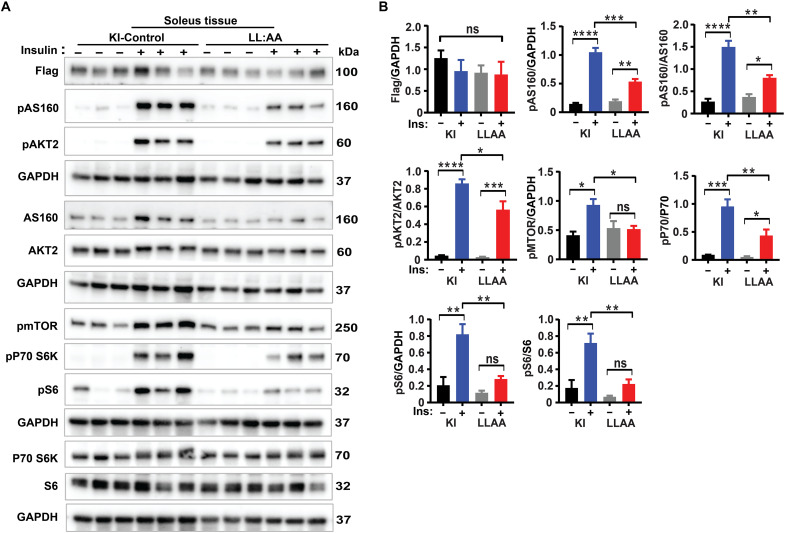
Lysosomal LRRC8A depletion impairs skeletal muscle insulin-PI3K-mTOR in vivo. (**A**) Western blots of Flag, p-AS160, AS160, p-AKT2, AKT2, p-mTOR, p-P70 S6K, P70 S6K, p-S6, S6, and GAPDH in soleus muscle from LRRC8A-3×Flag KI and LL:AA-3×Flag KI mice following in vivo insulin stimulation (5 μl from 100 IU/ml Humulin R insulin via inferior vena cava) for 10 min. (**B**) Densitometry quantification of (A). Statistical significance between the indicated group calculated with one-way ANOVA, Tukey’s multiple comparisons test. Error bars represent mean ± SEM. *P < 0.05, **P < 0.01, ***P < 0.001, ****P < 0.0001. *n* = 3 independent experiments.

In vitro experiments using primary muscle cells from LL:AA mice (Fig. 6, F and G) and in vivo hyperinsulinemic clamp studies (Fig. 8, H to Q) in LL:AA mice reveal that lysosomal LRRC8 channel depletion in skeletal muscle impairs insulin-PI3K-AKT signaling in vitro and systemic insulin sensitivity in vivo. Next, to examine insulin-PI3K-AKT-mTOR signaling in native muscle tissue of LL:AA KI mice, we conducted in vivo insulin stimulation in LL:AA KI and WT LRRC8A-3×Flag-KI-control mice after a 16-hour fast. Immunoblot results from soleus muscle reveal that PI3K (pAKT2 and pAS160) and mTOR (pMTOR, pP70 S6K, and pS6) signaling proteins are significantly reduced in the LL:AA mice as compared to WT LRRC8A-3×Flag-KI mice upon insulin stimulation (Fig. 9, A and B). These data are consistent with hyperinsulinemic clamp results that show reduced glucose uptake in soleus muscle of LL:AA mice and demonstrate that impaired skeletal muscle insulin signaling is contributing to systemic insulin resistance in mice with lysosomal LRRC8 depletion (Fig. 8L).

## DISCUSSION

Lysosomes regulate various cellular processes such as signaling, nutrient sensing, autophagy, and phagosome formation. Our findings reveal that LRRC8A forms a functional lysosomal channel complex, which regulates lysosomal morphology, pH, and LAMP levels. LRRC8A depletion leads to the formation of enlarged autophagosomes. In addition, there is evidence of impaired autophagic flux despite a lower lysosomal pH, suggesting that endocytosed material is unable to fuse with lysosomes or, even if they do fuse to form autolysosomes, the lysosomal pH is not optimal for the resident hydrolytic enzymes, resulting in accumulation of undigested material within the autophagosomes. To ensure proper enzymatic function of lysosomal hydrolases, an optimal pH range of 4.5 to 5.5 is essential within lysosomes. This pH balance is regulated by a variety of ion channels and pumps located on the lysosomal or endosomal membrane, including V-ATPase (*42*), TMEM175 (*43*), TMEM206 (PAC) (*12*), and ClC-3-7 (acting as a 2Cl^−^/1H^+^ antiporter) (*44*). It is also possible that depletion of LRRC8A protein could directly or indirectly alter the expression or canonical function of these proteins (V-ATPase, TMEM175, and ClC-3-7), resulting in deviations from optimal lysosomal pH, altered fusion of autophagosomes with lysosomes, and, ultimately, compromised autophagic flux. To maintain an acidic lysosomal lumen or high proton gradient, the permeation of an additional negatively charged counterion, such as Cl^−^, is necessary. This transport is facilitated by CLC7, which is present in the late endosome and lysosome (*45*). Notably, the presence of gain-of-function mutations in CLC7 (*46*) or the depletion of cellular PIP2 (*44*) can cause an elevation in the chloride (Cl^−^) gradient within the lysosomal lumen, resulting in hyperacidic lysosomes. We hypothesize that the Lyso-LRRC8A channel could act as a brake to prevent excessive acidification of the lysosome by facilitating the release of chloride ions (Cl^−^) from the lysosomal lumen, similar to the endosomal PAC channel (*12*). In addition, an alternative hypothesis arises from a recent publication by Zhang *et al.* (*43*), which demonstrated that lysosomal membrane proteins LAMP1 and LAMP2 directly bind to and allosterically inhibit the function of the pH-activated proton channel, TMEM175, facilitating a reduction in lysosomal pH necessary for proper lysosomal function. On the basis of these findings, it is possible that increased LAMP proteins in LRRC8A KO or lysosomal LRRC8-depleted LL:AA cells could potentially bind and allosterically inhibit lysosomal TMEM175 proton channel function, thereby lowering lysosomal pH.

While the mechanism underlying LAMP protein induction in LRRC8A KO or LL:AA cells is not yet determined, it is plausible that this up-regulation may result from aggregated dysfunctional lysosomes or possibly altered expression of genes involved in lysosomal biogenesis, such as *TFEB* and *TFE3*. LL:AA myotube RNA-seq transcript data reveal increased lysosomal hydrolase expression (such as cathepsin and lysozyme) as well as genes associated with the complement cascade—changes also observed in LRRC8A KO myotubes RNA-seq data (*20*). This suggests that abnormal protease activation in LL:AA cells could potentially trigger the activation of various complement-associated genes through site-specific cleavage. This activation may contribute to the development of autoimmune diseases and other pathological conditions. Furthermore, a recent study (*47*) also showed that dysregulation of lysosomal cathepsin expression leads to the permeabilization of lysosomal membranes. This, in turn, triggers inflammasome activation, accumulation of autophagosomes, defects in autophagy, and excessive lipid accumulation in essential metabolic tissues like adipose tissue, liver, and muscle tissue (*47*). These changes can contribute to obesity-related pathologies such as type 2 diabetes (T2D), fatty liver disease, and heart disease (*47*). These findings are also consistent with IPA pathway analysis indicating that PTEN, PI3K-AKT, and IGF signaling pathways and lysosome-associated functions are altered in LL:AA myotubes.

On the basis of our in vitro and in vivo data, LL:AA primary myotubes and skeletal muscle tissue phenocopy LRRC8A-null myotubes with respect to defective insulin-PI3K-AKT signaling, dysfunctional lysosomes, and alterations in metabolic pathways. These cumulative changes ultimately may lead to altered systemic metabolism and adiposity in vivo, with impaired glucose tolerance and insulin sensitivity and increased adiposity observed in LL:AA mice over time. However, there is no significant difference in overall lean muscle mass. These results are consistent with our previous findings, in which skeletal muscle–specific LRRC8A-null mice exhibit increased fat mass and insulin resistance, with no apparent difference in lean muscle mass (*20*), and the excess circulating glucose is redistributed to other metabolic tissues, such as adipose tissue and liver. LL:AA mice maintain functional LRRC8 channel activity at the plasma membrane with depleted lysosomal LRRC8A protein, and yet show abnormalities in systemic metabolism and skeletal muscle insulin signaling, pointing to lysosomal LRRC8 as a regulator of insulin action and systemic metabolism.

Numerous lines of evidence suggest that dysfunctional lysosomes, increased autophagosomes and impaired autophagy are significant contributors to the development of insulin resistance in both obesity and T2D (*47*–*49*). It has been proposed that reduced lysosomal pH decreases binding affinity of insulin to its receptor (IR) and enhances the dissociation rate of the insulin-bound IR complex within endocytosed lysosomes (*50*, *51*). Consistent with this proposed mechanism, our lysosomal pH rescue experiments in LRRC8A KO myotubes using lysosome-alkalinizing agents HCQ and Baf A1 increased lysosomal pH and restored defective insulin-PI3K signaling in LRRC8A KO cells. This suggests that maintaining an optimal lysosomal pH is crucial for regulating the insulin signaling, insulin sensitivity, and systemic glucose homeostasis. It is plausible that the process of endocytosis of the insulin receptor and its bound ligand (insulin) and internalization into catabolic active lysosomes for degradation occurs more rapidly in LL:AA myotubes. This could be attributed to either higher expression of cathepsin enzymes (*52*) or the increased acidic nature of the lysosomes, which favors more rapid dissociation of insulin from the insulin receptor (*35*) in LL:AA myotubes, thereby terminating insulin signaling and contributing to insulin resistance. In summary, our study provides evidence that an LRRC8 channel complex is present in a subset of lysosomes and regulates lysosome morphology, pH, autophagic flux, and PI3K-AKT-mTOR signaling. However, the stoichiometry and the presence of different LRRC8 subunits within the lysosomal LRRC8 complex still require further detailed investigation to understand their physiological significance in the context of lysosomal biology. In vivo experiments highlight a unique physiological function of the lysosomal LRRC8 complex in maintaining glucose homeostasis, insulin sensitivity, and adiposity under basal conditions.

## MATERIALS AND METHODS

A list of all antibodies, vectors, cell lines, reagents, and materials used in this study is provided in table S3.

### Animals

The Institutional Animal Care and Use Committee of Washington University in St. Louis approved all experimental procedures involving mice. All mice were housed in a temperature, humidity, and light-controlled room and allowed free access to water and food. Study mice were gender/age matched, and both male and female mice were used. C57BL/6N mice were obtained from Charles River Laboratories (Wilmington, MA, USA). All mice were fed ad libitum with regular chow (RC; NIH31 irradiated, #7913) diet.

The generation of the LRRC8A^fl/fl^ conditional mouse was described previously (*19*). LRRC8A-3×Flag-KI (KI) mice were generated by CRISPR-Cas9 gene editing where a 3×Flag epitope was placed at the C terminus of the *Lrrc8a* gene at the Mouse Genetics Core (MGC facility, Washington University, St. Louis, MO). LRRC8A-L706A;L707A-3×Flag-KI (LL:AA) mice were generated by CRISPR-Cas9 gene editing where L706;L707 dileucines were mutated to alanines on the background of the previously generated LRRC8A-3×Flag-KI mouse to generate mice with LRRC8A-L706A;L707A-3×Flag KI mutations. LoxP-STOP-LoxP-LAMP1-RFP-1×Flag-TEV-HA (LAMP1-RFP-Flag-HA) (*53*) conditional mice were generated in the laboratory of A. Diwan (Washington University, St. Louis, MO). Furthermore, LRRC8A-L706A;L707A-3×Flag-KI (LL:AA) mice were crossed with LoxP-STOP-LoxP-LAMP1-RFP-1×Flag-TEV-HA(LAMP1-RFP-Flag-HA) to generate LRRC8A-L706A;L707A-3×Flag-KI;LoxP-STOP-LoxP-LAMP1-RFP-1×Flag-TEV-HA (LL:AA-LAMP1-RFP-Flag-HA) mice.

### Adenovirus/plasmids

Adenovirus type 5 with Ad5-CMV-eGFP [3 × 10^10^ plaque-forming units (PFU)/ml], Ad5-CMV-Cre-eGFP (8 × 10^10^ PFU/ml), Ad5-U6-scramble-mCherry (sh-SCR) (9 × 10^10^ PFU/ml), and Ad5-mCherry-U6-shLRRC8A (sh LRRC8A) (1 × 10^11^ PFU/ml) were obtained from the University of Iowa Viral Vector Core. Ad5-mPGK-mLrrc8a-(L706A)(L707A)-3×Flag (LL:AA) (1 × 10^10^ PFU/ml), Ad5-mPGK-mLrrc8a-3×Flag (1 × 10^10^ PFU/ml), and Ad(RGD)-CMV-iCre (1 × 10^10^ PFU/ml) viruses were obtained from Vector Biolabs (Malvern, PA, USA). LRRC8C-P2A-mCherry and LRRC8A-L706A;L707A-ALFA (LL:AA-ALFA) plasmids were obtained from Vector Biolabs. LRRC8A-ALFA-IRES-EGFP and LRRC8A-ALFA plasmids were generated by S. G. Brohawn’s lab, University of California Berkeley (Berkeley, CA, USA).

### Electrophysiology

Patch-clamp recordings were performed in the whole-cell configuration at room temperature (RT), as described previously (*19*, *20*, *54*). Briefly, currents were recorded using either an Axopatch 200B amplifier or a MultiClamp 700B amplifier (Molecular Devices), both paired with a Digidata 1550 digitizer and data were acquired by pClamp 10.4 software. The extracellular solution for hypotonic stimulation contained 90 mM NaCl, 2 mM CsCl, 1 mM MgCl_2_, 1 mM CaCl_2_, 10 mM Hepes, and 10 mM mannitol, pH 7.4, with NaOH (210 mosmol/kg). The isotonic extracellular solution had the same composition as above, except that it contained 110 mM mannitol instead of 10 mM, resulting in an osmolarity of 300 mosmol/kg. The intracellular solution contained 120 mM l-aspartic acid, 20 mM CsCl, 1 mM MgCl_2_, 5 mM EGTA, 10 mM Hepes, 5 mM MgATP, 120 mM CsOH, and 0.1 mM GTP, pH 7.2, with CsOH and had an osmolarity of 280 to 290 mosmol/kg. The osmolarity was checked by a vapor pressure osmometer 5500 (Wescor). Currents were filtered at 10 kHz and sampled at 100-μs intervals. The patch pipettes were pulled from borosilicate glass capillary tubes (WPI) using a P-87 micropipette puller (Sutter Instruments). The pipette resistance was ~2 to 5 megohms when the patch pipette was filled with intracellular solution. The holding potential was 0 mV. Voltage ramps from −100 to +100 mV (at 0.4 mV/ms) were applied every 4 s. Clampfit 10 (Molecular Devices) was used for data analysis. Currents were normalized by cell capacitance to calculate current densities.

### Primary muscle satellite cell isolation

Satellite cell isolation and differentiation were performed as described previously (*20*). Briefly, gastrocnemius, tibialis, soleus, and quadriceps muscles were excised from mice (8 to 10 weeks old) and washed twice with cold 1× phosphate-buffered saline (PBS) supplemented with 1% penicillin-streptomycin (#15140–122, Gibco) and amphotericin B (300 μl/100 ml) (#30-003-CF, Corning, amphotericin B). The excised muscle tissue was incubated in Dulbecco’s modified Eagle’s medium (DMEM)–F12 media supplemented with collagenase II (2 mg/ml) (#17101-015, Gibco), 1% penicillin-streptomycin, and amphotericin B (300 μl/100 ml) in a 37°C water bath for 15 min, and subsequently incubated in a shaker (220 rpm) for 90 min at 37°C. Tissue was washed with warm 1× PBS supplemented with 1% penicillin-streptomycin and fungizone (300 μl/100 ml) and incubated again with DMEM-F12 media supplemented with collagenase II (1 mg/ml), dispase (0.5 mg/ml) (#61206900, Roche), 1% penicillin-streptomycin, and fungizone (300 μl/100 ml) in a shaker for 30 min at 37°C. Subsequently, the tissue was minced and passed through a cell strainer (100 μm), and after centrifugation, satellite cells were plated on Matrigel-coated dishes. The isolated satellite cells were stimulated to differentiate into myoblasts in DMEM-F12, 20% fetal bovine serum (FBS), basic fibroblast growth factor (bfgf; 40 ng/ml; Gibco #13256029), 1× nonessential amino acids, 0.14 mM β-mercaptoethanol, 1× penicillin/streptomycin, and amphotericin B. Myoblasts were maintained with bfgf (10 ng/ml) and then differentiated in DMEM-F12, 2% FBS, and 1× insulin–transferrin–selenium, when 70 to 80% confluency was reached.

### Cell culture and signaling studies

WT C2C12 and LRRC8A KO C2C12 cell lines were cultured at 37°C, 5% CO_2_ in DMEM (Gibco) supplemented with 10% FBS (Atlanta Bio) and antibiotics (1% penicillin-streptomycin). Cells were grown to 80% confluency and then transferred to differentiation media DMEM supplemented with antibiotics and 2% horse serum (#16050122, Gibco) to induce differentiation. The differentiation medium was changed every 2 days. Cells were allowed to differentiate into myotubes for 7 to 8 days. For leucine stimulation, differentiated C2C12 myotubes were incubated with Krebs-Ringer Bicarbonate Hepes (KRBH) buffer [129 mM NaCl, 5 mM NaHCO_3_, 4.8 mM KCl, 1.2 mM KH_2_PO_4_, 2.5 mM CaCl_2_, 2.4 mM MgSO_4_, 10 mM Hepes, 30 mM mannitol, and 0.1% bovine serum albumin (BSA), pH 7.4] without glucose for 3 hours. Subsequently, cells were stimulated either with glucose alone (129 mM NaCl, 5 mM NaHCO_3_, 4.8 mM KCl, 1.2 mM KH_2_PO_4_, 2.5 mM CaCl_2_, 2.4 mM MgSO_4_, 10 mM Hepes, 30 mM mannitol, and 0.1% BSA, pH 7.4) or without glucose for 3 hours. Subsequently cells were stimulated either with glucose alone (129 mM NaCl, 5 mM NaHCO_3_, 4.8 mM KCl, 1.2 mM KH_2_PO_4_, 2.5 mM CaCl_2_, 2.4 mM MgSO_4_, 10 mM Hepes, 24.5 mM mannitol, 5.5 mM glucose, and 0.1% BSA, pH 7.4) or glucose + leucine (129 mM NaCl, 5 mM NaHCO_3_, 4.8 mM KCl, 1.2 mM KH_2_PO_4_, 2.5 mM CaCl_2_, 2.4 mM MgSO_4_, 10 mM Hepes, 19.5 mM mannitol, 5.5 mM glucose, 5 mM leucine, and 0.1% BSA, pH 7.4) for 15 min. The osmolarity of the Krebs buffer was maintained at 300 mosmol by changing mannitol concentration. To perform leucine stimulation in LRRC8A KO primary muscle cells, satellite cells were isolated from LRRC8A^fl/fl^ mice and transduced with Ad5-CMV-eGFP or Ad5-CMV-Cre-eGFP [MOI (Multiplicity of Infection) 50 to 60] for 48 hours in differentiation media. After 3 days of differentiation, leucine stimulation was performed as described above. To examine leucine stimulation in LRRC8A KD of postdifferentiated WT C2C12 cells, we first differentiated WT C2C12 cell in differentiation for 6 days, subsequently transduced with Ad5-U6-scramble-mCherry (sh-SCR) or Ad5-mCherry-U6-shLRRC8A (sh LRRC8A) (KD) (MOI 50 to 60) for 48 hours, and allowed them to differentiate one additional day before using them for leucine stimulation as described above.

For insulin stimulation, differentiated primary myotubes were incubated in serum-free media (DMEM-F12, 1% penicillin-streptomycin) for 3 hours and stimulated with 0 and 10 nM insulin (#128-100, Cell Applications) in differentiation media (DMEM-F12, 2% FBS, 1× insulin-transferrin-selenium) for 15 min. To perform intracellular signaling in C2C12 WT and LRRC8A KO C2C12 myotubes, we reexpressed LRRC8A-3×Flag (Ad5-mPGK-mLrrc8a-3×Flag) and LRRC8A-L706A;L707A-3×Flag [Ad5-mPGK-mLrrc8a-(L706A)(L707A)-3×Flag] by transduction (MOI 80 to 100) in LRRC8A KO C2C12 cells in differentiation media (DMEM, 2% horse serum and 1% penicillin-streptomycin) for 48 hours and allowed them to further differentiate in differentiation media for 7 to 8 days. Differentiated myotubes were harvested in radioimmunoprecipitation assay (RIPA) buffer at basal condition for further signaling studies. To perform basal cellular signaling in primary skeletal muscle cells, LRRC8A-3×Flag-KI and LRRC8A-L706A; L707A-3×Flag KI cells were differentiated in differentiation media (DMEM-F12, 2% FBS, and 1× insulin-transferrin-selenium) for 3 days and myotubes were harvested in RIPA buffer.

For HCQ/Baf A1 treatment and insulin-stimulated signaling experiment, differentiated C2C12 WT and LRRC8A KO C2C12 cells were treated with HCQ (25 μM) or Baf A1 (10 nM) for 4 hours in differentiation media, without starvation. Subsequently, cells were stimulated with insulin (10 nM) for 15 min in the differentiation media supplemented with HCQ (25 μM) or Baf A1 (10 nM) before collecting lysates.

### Immunofluorescence, Pearson’s *R* quantification, and LysoTracker imaging

LRRC8A KO cells were cultured on coverslips and transfected with LRRC8A-ALFA or LL:AA-ALFA plasmids by using lipofectamine 2000 (#11668-019, Thermo Fisher Scientific) as per the manufacturer’s instructions. After 48 hours of transfection, cells were fixed with 2% paraformaldehyde (PFA) (#J19943-K2, Thermo Fisher Scientific) for 15 min in an incubator. Subsequently, fixed cells were washed three times with 1× PBS and permeabilized with 0.1% Triton X-100 (0.1% in 1× PBS) for 3 min at RT. Cells were washed three times with 1× PBS, and blocking was performed with 3% (w/v) BSA and 1% (w/v) milk mixture in PBST (0.1% Tween 20 in 1× PBS) for 1 hour at RT. Further, cells were incubated with primary antibody of anti-ALFA (1:250) and anti-LAMP1 (1:250) in blocking buffer overnight at 4°C. Cells were then washed three times with PBST and mounted on a glass slide with ProLong Diamond (#36980, Invitrogen) anti-fading media. All images were captured by using a STEDYCON Abberior microscope in a confocal and STED mode with 100× objective. The acquired images were deconvoluted by using Huygens software.

The Pearson’s colocalization coefficient was obtained using the JACoP plugin in ImageJ. All acquired images were converted to 16-bit images, and composite images were split into two color images. Auto-thresholding of images was applied by using the check option in the JACoP plugin and then hit analyze to obtain the Pearson colocalization value. For colocalization analysis, the rectangular box tool was used to crop selected areas from individual cells or from different frames of images within the Z-stacking images.

To perform the live-cell staining of LRRC8A-ALFA and LysoTracker Red, LRRC8A-ALFA was transiently transfected into LRRC8A KO C2C12 myoblasts. After 48 hours, live cells were pulsed with anti-ALFA-Atto643 antibody (1:100) for 5 min. Subsequently, the cells were replaced with fresh media and chased for 2 hours to allow the LRRC8A-ALFA–bound antibody to undergo endocytosis. Further labeling with LysoTracker Red (#70083, Biotium, LysoView 550) was performed in the same cell culture media for 15 min. The cells were imaged by using STEDYCON Abberior microscope in a confocal mode with 100× objective. The fluorescent intensities of LRRC8A-ALFA and LysoTracker Red were measured using the line scanning tool in ImageJ.

### pH measurement

Cells were grown on live-cell dishes for LysoSensor Yellow/Blue DND-160 (#7545, Thermo Fisher Scientific) ratiometric imaging, as described earlier (*55*, *56*). For some experiments, myoblasts were differentiated into myotubes and a freshly prepared Lysosensor (1 μM) was added to growth medium for 5 min (C2C12 cells) or 20 min (Primary muscle myotube) at 37°C. To obtain a pH standard curve, after Lysosensor labeling, cells were washed with Hanks’ balanced salt solution (HBSS; #14025-092) and replaced with standard pH buffer (140 mM KCl, 5 mM glucose, 1 mM CaCl_2_.2H_2_O, 1 mM Mgcl_2_, 20 mM Hepes, 20 mM MES, and 20 mM acetic acid, pH 3 to 7) supplemented with 20 μM nigericin and ratiometric imaging was performed. In a subset of experiments, differentiated myotubes were treated with Baf A1 (10 or 250 nM) and HCQ (25 μM) for 4 hours before labeling with Lysosensor. To reduce background fluorescence, imaging was performed in HBSS buffer. Lysosensor-stained cells were excited sequentially at 340 and 380 nm, and the resulting emissions were collected using a Fura-2 dichroic filter cube. Images were saved as TIFF files. The background was estimated using a rolling ball estimation method with a radius of 50 pixels in ImageJ. Following background subtraction, the intensity of the acquired field of view at 340 and 380 nm was measured using the “Measure” feature in ImageJ and then converted into the 340/380 ratio. Lysosomal pH for each field of view was determined using nonlinear least squares fit to the pH calibration curve. Typically, five to nine images were collected for each dish to measure ratiometric intensity. The scatter dot plot shows the representative lysosomal pH for all measurements from the acquired frame. Ratiometric images for lysosomal pH were obtained by using ImageJ. Briefly, 16-bit captured images of Ex340/380 were open, and after subtracting background, image intensities were divided by using process and calculator plus option in ImageJ. Lysosensor fluoresces brighter in the longer wavelength channel (380 nm) in lower pH (acidic) conditions, whereas at higher pH, the emission shifts to shorter wavelengths (340 nm). Therefore, the 340/380 ratio is a good measure of pH of the organelle. The 340/380 ratio was plotted as a function of condition and one-way analysis of variance (ANOVA) was used to estimate the significance of difference between different conditions.

### TEM imaging and lysosome quantification

TEM imaging and lysosome quantification were performed as described previously (*57*, *58*). Briefly, cells were grown on coverslips, and before fixation, the cell culture media was removed. Cells were washed with a washing buffer (0.15 M cacodylate buffer with 2 mM CaCl_2_) to remove any remaining cell culture media. Subsequently, cell fixation was performed with a fixative solution (2.5% glutaraldehyde, 2% paraformaldehyde, 0.15 M cacodylate buffer, and 2 mM CaCl_2_, pH 7.4) in an incubator for 15 s. The fixation was then continued overnight at RT on a shaker with gentle agitation. Samples were secondarily fixed in 1% osmium tetroxide and 1.5% potassium ferrocyanide in 0.1 M sodium cacodylate buffer for 30 min to 1 hour after an initial fixation and rinse. Following secondary fixation, samples were washed in 0.1 M sodium cacodylate buffer (pH 7.3) and diH_2_O, then incubated overnight at 4°C in 2.5% uranyl acetate. Dehydration was performed using an ethanol gradient, followed by infiltration with Eponate 12. Samples were cured at 70°C overnight, and sections were prepared using an ultramicrotome and counterstained with uranyl acetate and Reynold’s lead citrate. TEM images were acquired using either a JEOL JEM-1230 (120 kV) or a JEOL 1400 (80 kV) transmission electron microscope (JEOL JEM-1400 Plus, 120 kV). Lysosome area and circularity were quantified by ImageJ as described previously (*57*, *58*). Briefly, images were uploaded in TIFF format and divided into four quadrants using the ImageJ quadrant picking plugin. Two quadrants were randomly selected for detailed analysis. Three independent, blinded analysts quantified lysosomes and autophagosomes, averaging their results to minimize bias. A minimum of 7 to 10 cells were analyzed per sample to ensure reproducibility. Lysosome area and circularity were obtained by manual tracing using the freehand tool in NIH ImageJ.

### Lysosomal immunoprecipitation

Lyso-IP from tissues or cells were adapted from Abu-Remaileh *et al.* (*59*). Briefly, differentiated myotubes were washed with cold 1× PBS, and scraped in 2 ml of homogenization buffer (50 mM potassium chloride, 90 mM potassium gluconate, 1 mM EGTA, 50 mM sucrose, 20 mM Hepes, and 5 mM glucose, pH 7.4, supplemented with protease and phosphatase inhibitors). The lysate was passed through a 26-gauge syringe five to seven times and Dounce homogenized with six to eight strokes with a glass homogenizer under ice-cold conditions. The homogenized lysate was centrifuged at 6800*g* for 5 min at 4°C, and the postnuclear supernatant (PNS) fraction passed through a 70-μm preseparation filter (#130-095-823, Miltenyi Biotec) equilibrated with homogenization buffer. The filtered PNS fraction was transferred to 50 μl of anti-HA (#88836, Thermo Fisher Scientific) magnetic beads and rotated for 15 to 20 min in a cold room. The PNS-bound magnetic beads were separated by using a magnetic bead rack separator. The PNS-bound magnetic beads were washed three times with washing buffer (1× PBS, 2 mM EDTA, and 0.5% BSA, pH 7.4). After washing, half of the PNS-bound beads were Triton X-100 treated [150 mM NaCl, 40 mM Hepes, 2.5 mM MgCl_2_, 1% Triton X-100, and 2 mM EGTA, with protease/phosphatase inhibitors (Roche)] for 10 min on ice. After separating the supernatant, the beads were lysed by using 60 to 70 μl of lysis buffer. Subsequently, Triton X-100-treated supernatant and beads (pellet) were boiled with 4× Laemmli buffer.

To perform Lyso IP from mouse skeletal muscle (tibialis and soleus) or heart, the tissue was freshly dissected under a dissecting microscope and then washed with ice-cold PBS. The washed tissues were diced into small chunks using a razor blade and transferred to a cryotube with 500 μl of homogenization buffer (50 mM potassium chloride, 90 mM potassium gluconate, 1 mM EGTA, 50 mM sucrose, 20 mM Hepes, and 5 mM glucose, pH 7.4, supplemented with protease and phosphatase inhibitors) for further mechanical grinding using an IKA-T10 Basic Ultra-Turrax Disperser Homogenizer at maximum speed on ice. The lysate was transferred to a glass Dounce homogenizer to further homogenize the lysate with 20 to 30 strokes under ice-cold conditions. The homogenized lysate was transferred to a microcentrifuge tube, the volume was adjusted to 1 ml, and the lysate was centrifuged at 6800*g* for 5 min at 4°C. The supernatant or PNS was carefully transferred to a tube, and the volume was adjusted to 2 ml by adding homogenization buffer. The PNS fraction was passed through a 70-μm preseparation filter (#130-095-823, Miltenyi Biotec) equilibrated with homogenization buffer. The filtered PNS fraction was then transferred to 50 μl of anti-Flag microbeads (#130-101-591, Miltenyi Biotec, μMACS and MultiMACS DYKDDDDK Isolation Kits) and rotated for 15 min in a cold room. The PNS-bound beads were then filtered through a 20-μm preseparation filter (#130-101-812, Miltenyi Biotec) preequilibrated with homogenization buffer. The filtered PNS beads suspension was transferred to a MACS column on a MACS separator magnet and washed three times with 5 ml of wash buffer (1× PBS, 2 mM EDTA, and 0.5% BSA, pH 7.4), allowing it to drain by gravity flow. After washing, the column was removed from the MACS separator, and 2 ml of homogenization buffer was added to elute the tagged protein bound to the beads. The eluted sample was subsequently centrifuged at 20,000*g* for 15 min to pellet the beads with bound lysosomes. Furthermore, to perform Triton X-100 treatment, half of the beads were treated with 80 μl of lysis buffer (150 mM NaCl, 40 mM Hepes, 2.5 mM MgCl_2_, 1% Triton X-100, and 2 mM EGTA, with proteinase/phosphatase inhibitors) for 15 min and centrifuged at 20,000*g* for 15 min. The Triton X-100-treated supernatant and pellet (beads) were then boiled with 4× Laemmli buffer for WB analysis.

### RNA sequencing

Differentiated primary myotubes were solubilized in TRIzol, and the total RNA was isolated using a PureLink RNA kit (#12183018A, Thermo Fisher Scientific) and a column DNase digestion kit (#12185010, Thermo Fisher Scientific). The RNA quality analysis and sequencing was performed by Genome Engineering & Stem Cell Center (GESC@MGI) at the Washington University in St. Louis. Total RNA integrity was determined using Agilent Bioanalyzer or 4200 Tapestation. Library preparation was performed with 10 ng of total RNA with a Bioanalyzer RNA Integrity Number (RIN) score greater than 8.0. ds-cDNA was prepared using the SMARTer Ultra Low RNA kit for Illumina Sequencing (Takara-Clontech) as per the manufacturer’s protocol. cDNA was fragmented using a Covaris E220 sonicator using peak incident power 18, duty factor 20%, and cycles per burst 50 for 120 s. cDNA was blunt ended, had an A base added to the 3′ ends, and then had Illumina sequencing adapters ligated to the ends. Ligated fragments were then amplified for 12 to 15 cycles using primers incorporating unique dual index tags. Fragments were sequenced on an Illumina NovaSeq X Plus using paired-end reads extending 150 bases. Basecalls and demultiplexing were performed with Illumina’s DRAGEN and BCLconvert version 4.2.4 software. RNA-seq reads were then aligned to the Ensembl release 101 GRCm38 primary assembly with STAR version 2.7.9a. Gene counts were derived from the number of uniquely aligned unambiguous reads by Subread:featureCount version 2.0.3. Isoform expression of known Ensembl transcripts was quantified with Salmon version 1.5.2. Sequencing performance was assessed for the total number of aligned reads, total number of uniquely aligned reads, and features detected. The ribosomal fraction, known junction saturation, and read distribution over known gene models were quantified with RSeQC version 4.0.

All gene counts were then imported into the R/Bioconductor package EdgeR and Trimmed Mean of M-values (TMM) normalization size factors were calculated to adjust for samples for differences in library size. Ribosomal genes and genes not expressed in the smallest group size minus one samples greater than one count per million were excluded from further analysis. The TMM size factors and the matrix of counts were then imported into the R/Bioconductor package Limma. Weighted likelihoods based on the observed mean-variance relationship of every gene and sample were then calculated for all samples with the voomWithQualityWeights function and were fitted using a Limma generalized linear model with additional unknown latent effects as determined by surrogate variable analysis (SVA). The performance of all genes was assessed with plots of the residual SD of every gene to their average log count with a robustly fitted trend line of the residuals. Differential expression analysis was then performed to analyze for differences between conditions, and the results were filtered for only those genes with Benjamini-Hochberg false discovery rate adjusted *P* values less than or equal to 0.05.

Sequencing results were uploaded and analyzed using BaseSpace (Illumina). Sequences were trimmed to 125 bp using the FastQ Toolkit (version 2.2.5). RNA-seq alignment (version 1.1.1) aligned the sequences against the *Mus musculus* (UCSC mm10) genome. Transcript assembly and differential gene expression were determined by Cufflinks Assembly and DE (version 2.1.0). IPA (QIAGEN) was used to analyze significantly regulated genes using genes with >1.5-fold change in gene expression and a false discovery rate of <0.05. Heatmaps were generated using *z*-scores and plotted using GraphPad (version 10.2.1).

### Western blot

Cells were washed with ice-cold 1× PBS and lysed in ice-cold RIPA lysis buffer (150 mM NaCl, 20 mM Hepes, 1% NP-40, and 5 mM EDTA, pH 7.5) with added protease/phosphatase inhibitor (Roche). Cell lysates were further sonicated for two to three cycles (20% pulse frequency for 20 s) and centrifuged at 13,000 rpm for 20 min at 4°C. The supernatant was collected and estimated for protein concentration using DC protein assay kit (Bio-Rad). For soleus muscle, the tissue lysis protocol was used as described earlier with minor modifications (*60*). The tissue was homogenized in a TissueLyser LT (Qiagen, #85600) (50 Hz, 5 min) in 8 volumes of ice-cold muscle homogenization buffer (50 mM tris, 25 mM NaCl, 0.2% Nonidet P-40, 10 mM EGTA, 2.5 mM EDTA, 20 mM NaF, 25 mM Na_4_P_2_O_7_ · 10H_2_O, and 2 mM Na_3_VO_4_, pH 7.4) supplemented with protease/phosphatase inhibitor (Roche). The homogenized lysate of soleus was centrifuged two times at 18,500*g* for 20 min at 4°C and supernatant was collected for protein estimation.

For immunoblotting, an appropriate volume of 4× Laemmli (Bio-Rad) sample loading buffer was added to the sample (10 to 20 μg of protein), then heated at 95°C for 5 min before loading into 4 to 20% gel (Bio-Rad). Proteins were separated using running buffer (Bio-Rad) for 2 hours at 110 V. Proteins were transferred to polyvinylidene difluoride membrane (Bio-Rad) and membrane blocked in 5% (w/v) BSA or 5% (w/v) milk in TBST buffer (0.2 M tris, 1.37 M NaCl, and 0.2% Tween 20, pH 7.4) at RT for 1 hour. Blots were incubated with primary antibodies (1:1000) at 4°C overnight, followed by incubation with secondary antibody (Invitrogen, Goat-anti-Rat #31470, Goat-anti-mouse #170-5047, Rabbit-anti-mouse #D3V2A, Goat-anti-rabbit #170–6515, all used at 1:10000) at RT for 1 hour. Membranes were washed three times with TBST buffer and imaged by chemiluminescence (Pierce) by using a ChemiDoc imaging system (Bio-Rad). Images were further analyzed for band intensities using ImageJ software. β-Actin or GAPDH levels were quantified for equal protein loading.

### Metabolic phenotyping and in vivo insulin stimulation

Mouse body composition (fat and lean mass) was measured by nuclear magnetic resonance (NMR; Echo-MRI 3-in-1 analyzer, EchoMRI, LLC) as described previously (*20*). To perform the GTT, mice were fasted for 6 hours and administered glucose (1 g/kg body weight) by intraperitoneal injection. Glucose levels were monitored in the blood via tail bleeds using a glucometer (Bayer Healthcare LLC) at the indicated times. To perform the insulin tolerance test (ITT), mice were fasted for 4 hours, and after an intraperitoneal injection of insulin (HumulinR, 1 U/kg), glucose levels were measured by a glucometer at the indicated times.

For in vivo insulin stimulation, as previously described (*61*), mice were fasted for 16 hours and then anesthetized with 2% isoflurane. The vena cava was exposed, and the mice were injected with either insulin (5 μl, human insulin 100 IU/ml) or saline. After 10 min postinjection, the soleus muscle was snap frozen for further analysis.

### Hyperinsulinemic-euglycemic glucose clamps

A sterile silicone catheter was introduced into the jugular vein under isoflurane anesthesia. Mice were allowed to fully recover from surgery for 5 to 6 days before undergoing hyperinsulinemic-euglycemic clamp procedures. Animals showing impaired recovery (as evidenced by wound infection, weight loss of more than 10% compared to presurgery weight) were excluded from further experiments. Clamp procedures were performed in 5-hour fasted, unrestrained, conscious mice by using an infusion swivel (#375/D/22QM, Instech, Plymouth Meeting PA) to allow free movement. Whole-body glucose flux was traced by infusion of d-[3-^3^H]-glucose. After an 80-min basal sampling period, insulin administration was initiated with a bolus infusion (32.5 mU/kg) over 1 min followed by 3.25 mU/kg/min continuous infusion. Simultaneously, 50% dextrose in saline was infused, adjusting the rate every 10 min to achieve a goal blood glucose of 150 mg/dl. Once the desired blood glucose level was stable (typically after 70 to 110 min), a glucose uptake tracer (2-DG; Perkin Elmer, catalog no. NEC495001MC) was administered as a single bolus at a dose of 13 μCi over 1 min at 75 min. ^14^C-2-deoxy-d-glucose-6-phosphate tracer enrichment was used to measure glucose uptake into specific tissues. Blood samples (~20 to 25 μl) from tail-cut were collected at 80, 90, 100, 110 and 120 min and centrifuged, and plasma was transferred to fresh tubes and stored frozen at −80°C until use. After the clamp period, mice were anesthetized with isoflurane and euthanized, with tissues of interest subsequently harvested and frozen.

Glucose concentrations in the plasma and in the 50% dextrose infusates were measured by an enzymatic method (#GMD9, Analox Instruments Ltd., King William Street Amblecote Stourbridge). Plasma samples were treated with Ba(OH)_2_ and ZnSO_4_ and centrifuged at 16,000*g* for 5 min. The supernatant was dried overnight at 50°C, then reconstituted in distilled water. Diluted ^3^H (3)-glucose infusate was processed in the same manner as plasma samples to serve as reference. Tracer (^3^H and ^14^C) activity was measured using a liquid scintillation counter (Ultima Gold, Perkin Elmer, Waltham, MA). The obtained values were used for the determination of the relevant glucose turnover parameters (R_d_ and R_a_). Glucose appearance and disappearance rates were calculated using Steele’s equations (*62*). Small (~20 to 40 mg) tissue samples were treated with 0.5% perchloric acid, homogenized using a mechanical device (TissueLyzer, Qiagen, Hilden, Germany), then centrifuged at 4000*g* for 20 min. Supernatant was neutralized with KOH, then centrifuged at 10,000*g* for 10 min. A portion of the resulting supernatant underwent liquid scintillation counting to determine total tracer (^14^C) activity (both phosphorylated and nonphosphorylated 2-DG). A separate portion was treated with Ba(OH)_2_ and ZnSO_4,_ then centrifuged at 16,000*g* for 5 min. The resulting supernatant underwent liquid scintillation counting to determine nonphosphorylated 2-DG activity (^14^C). The difference between radioactivity in the first and second supernatant corresponds to the abundance of phosphorylated ^14^C 2-DG in the tissue, which was used to determine tissue-specific glucose uptake by dividing by the integrated area of the plasma 2-DG activity curve. Plasma insulin concentration was measured using a chemiluminescence ELISA (enzyme-linked immunosorbent assay) kit (#80-INSMR-CH01, Stellux, from ALPCO).

### Statistics

Data are presented as mean ± SEM or SD. Two-tailed paired or unpaired Student’s *t* tests were used for comparison between two groups. For three or more groups, data were analyzed by one-way ANOVA and Tukey’s post hoc test. For GTTs and ITTs, two-way ANOVA was used. A *P* value <0.05 was considered statistically significant. *, **, and *** represent *P* values less than 0.05, 0.01, and 0.001, respectively.

## Supplementary Materials

### The PDF file includes:

Figs. S1 to S9 Legends for tables S1 and S2 Table S3 Legend for movie S1 Data supporting WB figures References
