# Supplementary material for: Lysosomal LRRC8 complex impacts lysosomal pH, morphology, and systemic glucose metabolism
Source: Sci Adv. 2025 Sep 26;11(39):eadt6366. doi: 10.1126/sciadv.adt6366 (PMC12466849; doi:10.1126/sciadv.adt6366)
Supplement: Supplementary file 1 — Figs. S1 to S9 Legends for tables S1 and S2 Table S3 Legend for movie S1 Data supporting WB figures References [file sciadv.adt6366_sm.pdf]

Science Advances
AAAS

Supplementary Materials for

Lysosomal LRRC8 complex impacts lysosomal pH, morphology, and systemic glucose metabolism

Ashutosh Kumar *et al.*

Corresponding author: Rajan Sah, rajan.sah@wustl.edu

Sci. Adv. **11**, eadt6366 (2025)
DOI: 10.1126/sciadv.adt6366

The PDF file includes:

Figs. S1 to S9
Legends for tables S1 and S2
Table S3
Legend for movie S1
Data supporting WB figures
References

Other Supplementary Material for this manuscript includes the following:

Tables S1 and S2
Movie S1

figure S1

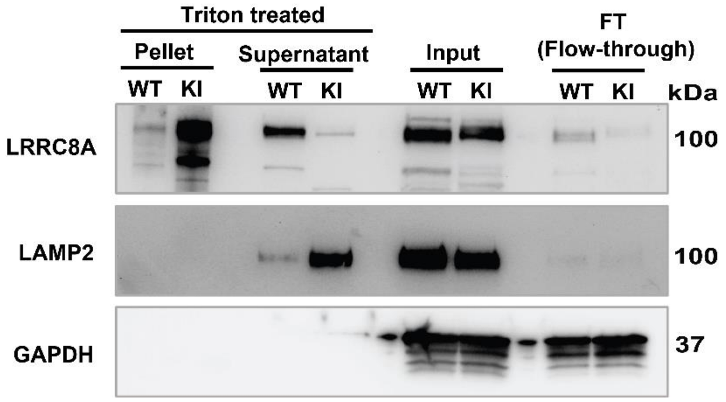

Figure S1. Immunoprecipitating endogenous LRRC8A containing lysosomes from cardiac tissue.

Organellar/Lysosomal IP performed on cardiac tissue of LRRC8A-3xFlag (KI) and WT control mice using anti-Flag magnetic beads. WB of lysosomal IP showing enriched LAMP2 protein in Triton-treated supernatant protein fraction of LRRC8A-3xFlag (KI) in comparison to WT. LRRC8A protein is enriched in the Triton-treated pellet fraction, as it is bound to anti-Flag magnetic beads. GAPDH served as loading control.

figure S2

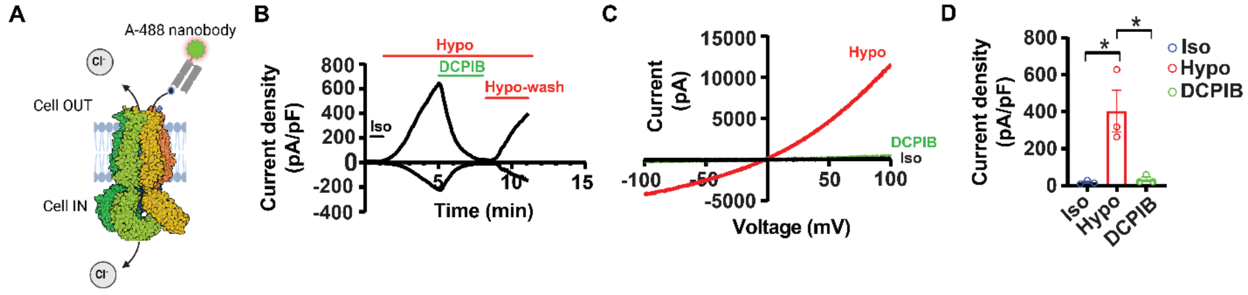

Figure S2. Functional characterization of LRRC8A-ALFA tagged protein in HeLa cells.

A, Cartoon depicting a LRRC8A heterohexameric structure showing ALFA epitope (13 aa) inserted within the first extracellular loop of mouse LRRC8A protein by replacing 77-90 aa. **B-D,** Whole-cell current density over time (B), current-voltage relationship (C) and mean current density at +100 mV (D) from 5 KO (LRRC8A/B/C/D/E KO) HeLa cells transiently co-transfected with LRRC8A-ALFA-IRES-EGFP and LRRC8C-P2A-mCherry. Voltage-ramp -100 to +100 mV were applied in presence/absence of isotonic (300 mOsm) and hypotonic (210 mOsm) solution and subsequent inhibition of VRAC current with 10 μM of DCPIB. statistical test for significance between the indicated values were calculated using a two-tailed Student's t-test. Error bars represent mean ± s.e.m. *, $P < 0.05$, **, $P < 0.01$, ***, $P < 0.001$, ****, $P < 0.0001$. n = 3, independent experiments. Figure **S2A** created in BioRender. Sah, R. (2025) https://BioRender.com/6hdznma

figure S3

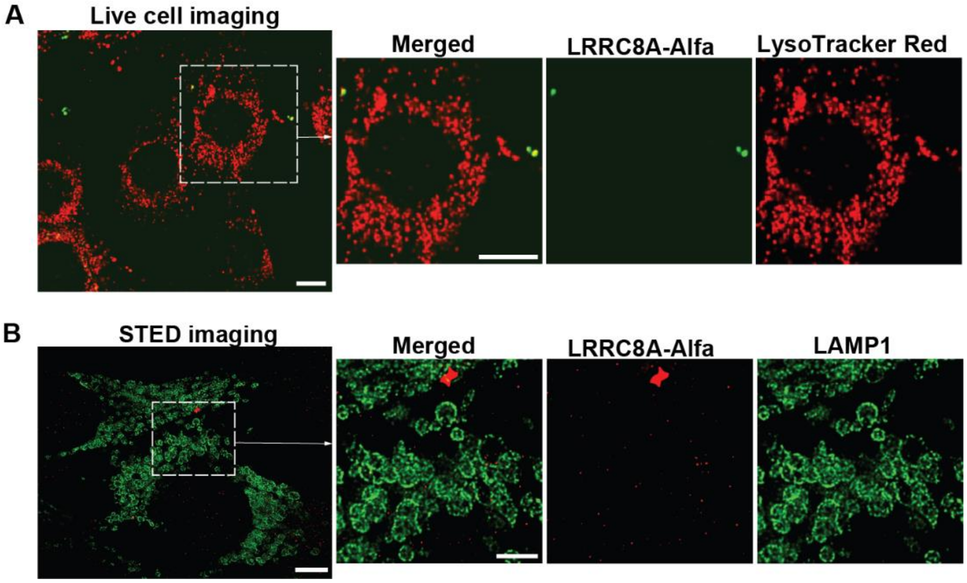

Figure S3. Confocal and Stimulated Emission Depletion (STED) immunofluorescence LRRC8A-ALFA imaging of untransfected LRRC8A KO C2C12 myoblasts.

A, Live cell confocal imaging of untransfected LRRC8A KO C2C12 myoblast stained with Lysotracker red, followed by a pulse with anti-ALFA-Alexa643 (Green, pseudo color) antibody for 5 minutes and chased for 2 hours. Scale bar: 10 µm. **B,** Stimulated Emission Depletion (STED) super resolution imaging of untransfected LRRC8A KO C2C12 myoblast immunostained with LRRC8A-ALFA (Red) and LAMP1 (Green) for lysosomes. Scale bar: 5 µm and 2 µm (inset).

figure S4

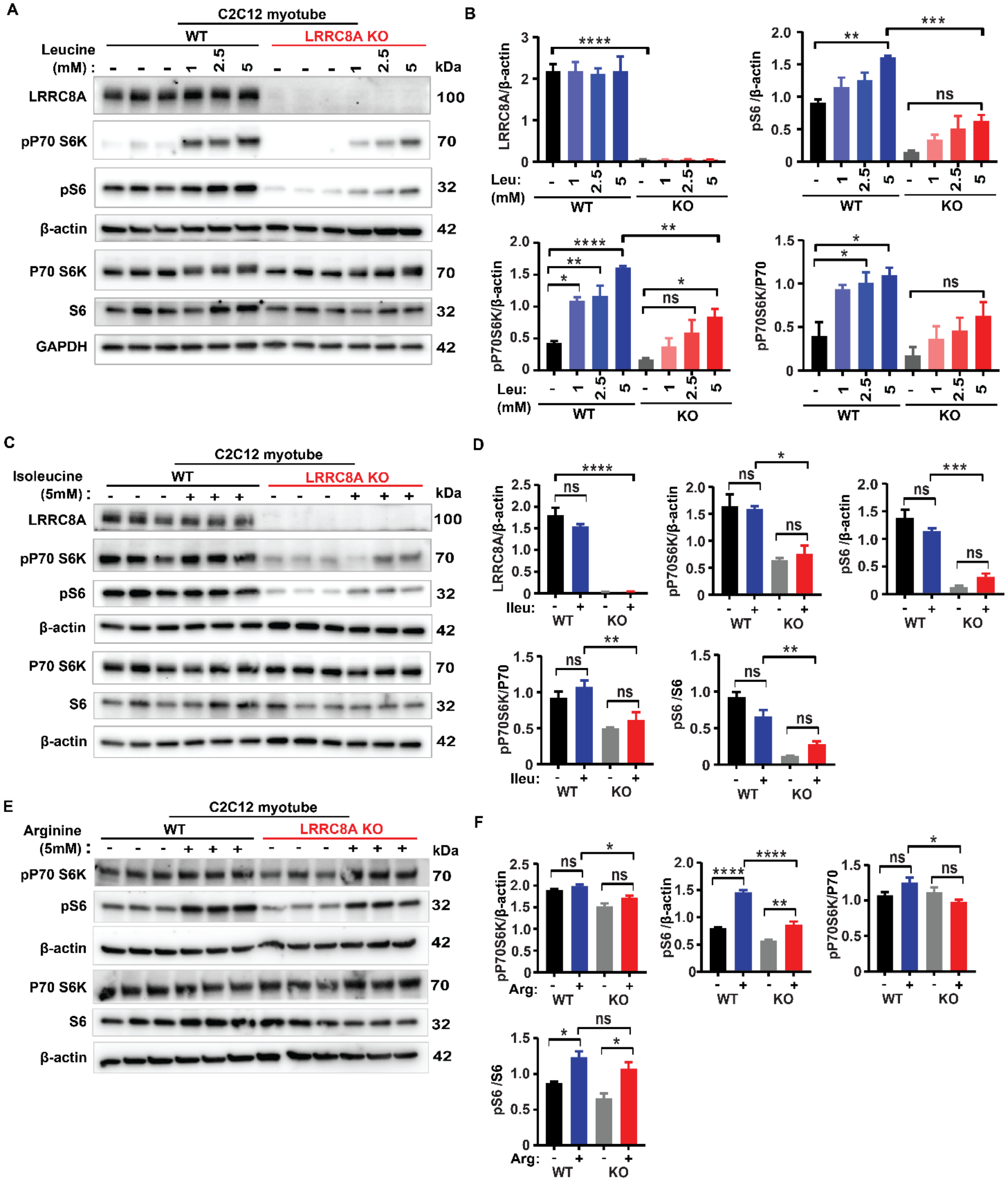
A
C2C12 myotube
WT
LRRC8A KO
Leucine
(mM) :
- - - 1 2.5 5 - - - 1 2.5 5
kDa
LRRC8A
100
pP70 S6K
70
pS6
32
β-actin
42
P70 S6K
70
S6
32
GAPDH
42
B
LRRC8A/β-actin
****
pS6 /β-actin
**
***
ns
pP70S6K/β-actin
****
**
**
*
ns
*
pP70S6K/P70
*
*
ns
Leu:
(mM)
WT
KO
C
C2C12 myotube
Isoleucine
(5mM) :
WT
LRRC8A KO
- - - + + + - - - + + +
kDa
LRRC8A
100
pP70 S6K
70
pS6
32
β-actin
42
P70 S6K
70
S6
32
β-actin
42
D
LRRC8A/β-actin
****
ns
pP70S6K/β-actin
*
ns
ns
pS6 /β-actin
***
ns
ns
pP70S6K/P70
**
ns
ns
pS6 /S6
**
ns
ns
Ileu:
WT
KO
E
C2C12 myotube
Arginine
(5mM) :
WT
LRRC8A KO
- - - + + + - - - + + +
kDa
pP70 S6K
70
pS6
32
β-actin
42
P70 S6K
70
S6
32
β-actin
42
F
pP70S6K/β-actin
*
ns
ns
pS6 /β-actin
****
****
**
pP70S6K/P70
*
ns
ns
pS6 /S6
ns
*
*
Arg:
WT
KO

Figure S4. Leucine, isoleucine and arginine stimulated mTOR signaling in WT and LRRC8A KO myotubes.

A, Western blot of LRRC8A, p-P70 S6k, P70 S6K, pS6, S6, and β-actin or GAPDH in WT and LRRC8A KO C2C12 myotubes show dose-dependent (1, 2.5 and 5 mM) leucine stimulation for 15 minutes. **B,** Densitometry quantification of leucine-stimulated signaling WB of (A). All experiments were performed in triplicate in three independent sets of experiments. **C,** WB of LRRC8A, β-actin, p-P70 S6K, P70 S6K, p-S6, S6 protein in WT and LRRC8A KO C2C12 myotubes after isoleucine (5 mM) stimulation for 15 minutes. **D,** Densitometry quantification of isoleucine-stimulated signaling WB of (C). **E,** WB of LRRC8A, β-actin, p-P70 S6K, P70 S6K, pS6, S6 protein in WT and LRRC8A KO C2C12 myotubes after arginine (5 mM) stimulation for 15 minutes. **F,** Densitometry quantification of arginine-stimulated signaling WB (E). Statistical significance between the indicated values were calculated using Statistical significance between the indicated group calculated with one-way Anova, Tukey's multiple comparisons test. Error bars represent mean ± s.e.m. *, $P < 0.05$, **, $P < 0.01$, ***, $P < 0.001$, ****, $P < 0.0001$. $n = 3$, independent experiments.

figure S5

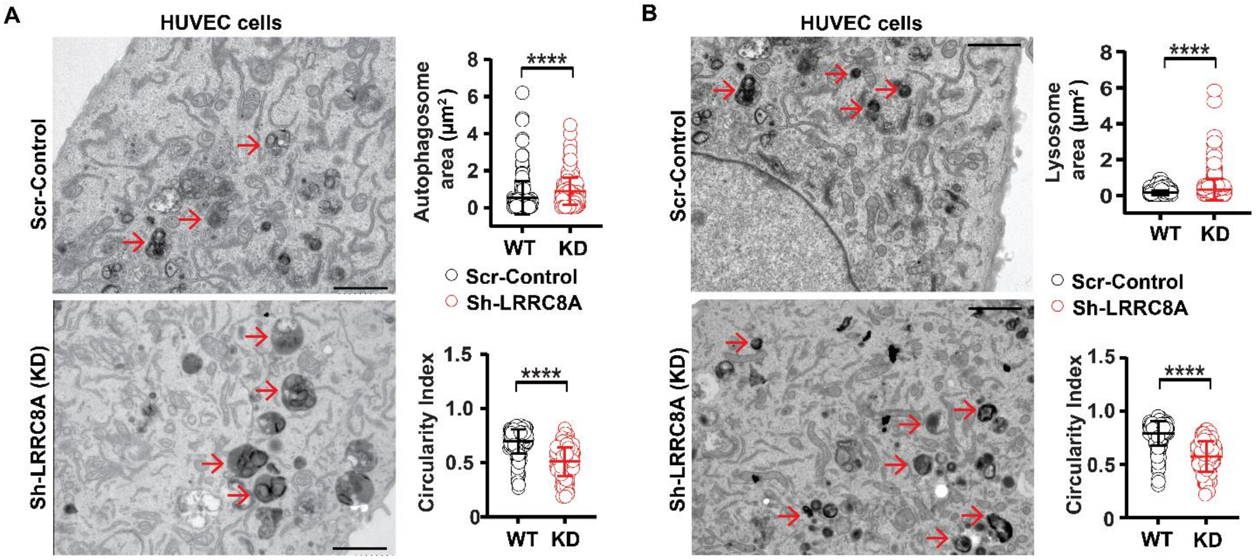

Figure S5. LRRC8A depleted human umbilical vein endothelial cells show enlarged lysosomes.

A&B, TEM images of WT human umbilical vein endothelial cell (HUVEC) and LRRC8A KD HUVEC showing enlarged autophagosome and lysosomes. The autophagosome area (WT=401, LRRC8A KD=586 autophagosomes), lysosome area (WT=497, LRRC8A KD=556 lysosomes) and circularity index for autophagosome (WT=419, LRRC8A KD=572 autophagosomes) and lysosomes (WT=254, LRRC8A KD=213 lysosomes) shown on the right side of images. Scale bar: 2 µm. Statistical significance between the indicated group in panel A and B were calculated by Mann-Whitney test. Error bars represent standard deviation (SD).

figure S6

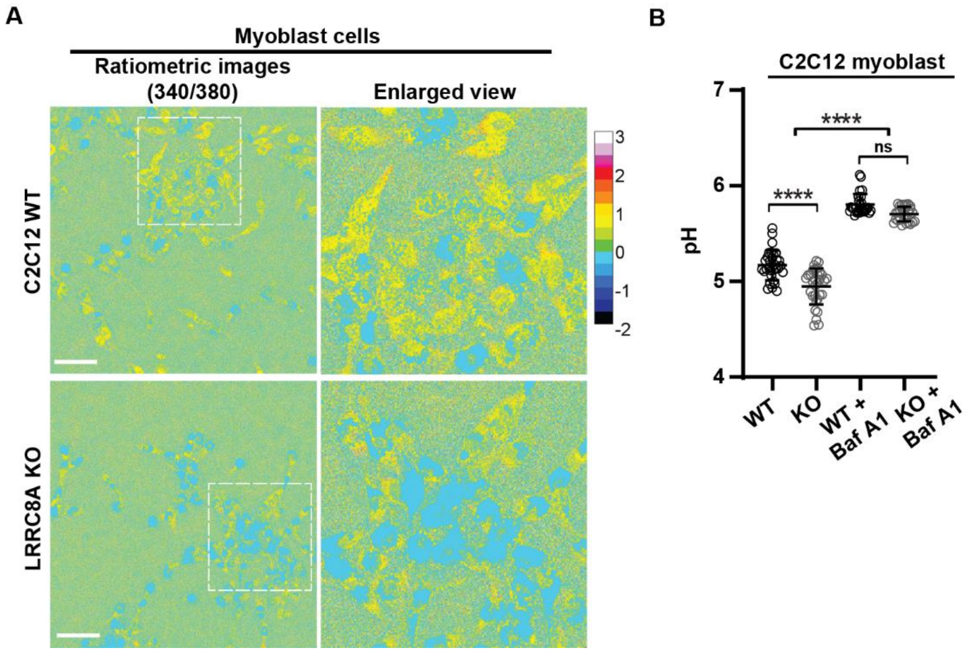

Figure S6. LRRC8A depletion decreases lysosomal pH in C2C12 myoblasts.

A, Fluorescence images of Lysosensor labeled images of WT and LRRC8A KO myoblasts. Scale bar: 100 µm. **B,** Lysosomal pH of WT, LRRC8A KO, WT + BafA1 and LRRC8A KO + BafA1 myoblasts which were determined from the non-linear least squares fit to the pH calibration curve. Scatter dot plot displays field of view quantification for 31 (WT), 31 (KO), 27 (WT + Baf A1) and 27 (KO + Baf A1), imaged from 2-3 dishes per condition. Statistical significance between the indicated values were calculated using one-way Anova, Tukey's multiple comparisons test. Error bars represent standard deviation (SD). *, $P < 0.05$, **, $P < 0.01$, ***, $P < 0.001$, ****, $P < 0.0001$.

figure S7

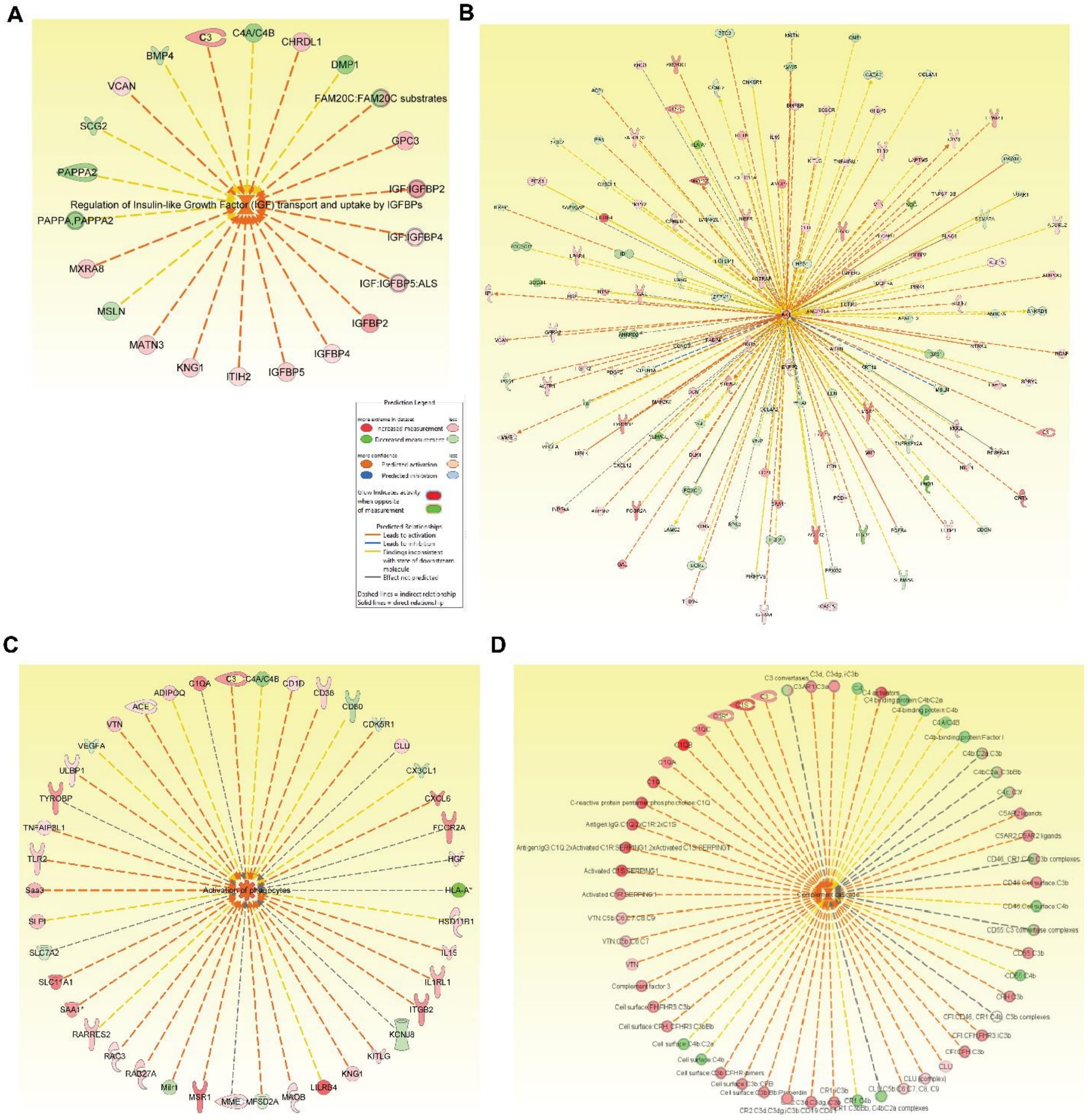

A
B
C
D
C3
C4A/C4B
CHRDL1
BMP4
DMP1
VCAN
FAM20C:FAM20C substrates
SCG2
GPC3
PAPPA2
IGF:IGFBP2
Regulation of Insulin-like Growth Factor (IGF) transport and uptake by IGFBPs
PAPPA,PAPPA2
IGF:IGFBP4
MXRA8
IGF:IGFBP5:ALS
MSLN
IGFBP2
MATN3
IGFBP4
KNG1
ITIH2
IGFBP5
Prediction Legend
Increased measurement
Decreased measurement
Predicted activation
Predicted inhibition
Predicted Relationships
Leads to activation
Leads to inhibition
Findings inconsistent with state of downstream molecule
Effect not predicted
Dashed lines = indirect relationship
Solid lines = direct relationship
C1QA
ADIPOQ
CD1D
CD36
ACE
CD80
VTN
CDK5R1
VEGFA
CLU
ULBP1
CX3CL1
TYROBP
CXCL6
TNFAIP8L1
FCGR2A
TLR2
HGF
Saa3
Activation of phagocytes
HLA-A*
SLPI
HSD11B1
SLC7A2
IL15
SLC11A1
IL1RL1
SAA1*
ITGB2
RARRES2
KCNJ8
RAC3
KITLG
RAB27A
KNG1
Milr1
MSR1
MME
MFSD2A
MAOB
LILRB4
C3 convertases
C3d, C3dg, iC3b
C4 activators
C4 binding protein:C4bC2a
C4-binding protein:C4b
C4A/C4B
C4b-binding protein:Factor I
C1QC
C1QB
C1QA
C1Q
C-reactive protein pentamer:phosphocholine:C1Q
C5AR2 ligands
C5AR2:C5AR2 ligands
CD46, CR1:C4b:C3b complexes
Activated C1S:SERPING1
CD46:Cell surface:C3b
Complement cascade
Activated C1R:SERPING1
CD46:Cell surface:C4b
CD55:C3 convertase complexes
VTN:C5b:C6:C7:C8:C9
VTN:C5b:C6:C7
VTN
Complement factor 3
Cell surface:C4b:C2a
Cell surface:C4b
Cell surface:C3b:CFHR dimers
Cell surface:C3b:CFB
Cell surface:C3b:Bb:Properdin
CLU

Figure S7. Interaction network analysis derived from RNA sequencing of lysosomal LRRC8A-depleted myotubes.

A-D Interaction networks identified affected cellular signaling pathway in LL:AA such as regulation of (A) Insulin-like growth factor transport and uptake, (B) AKT signaling, (C) activation of phagocytosis, (D) complements cascade. Network associated gene legends and symbol indicated by predicted legend figure.

figure S8

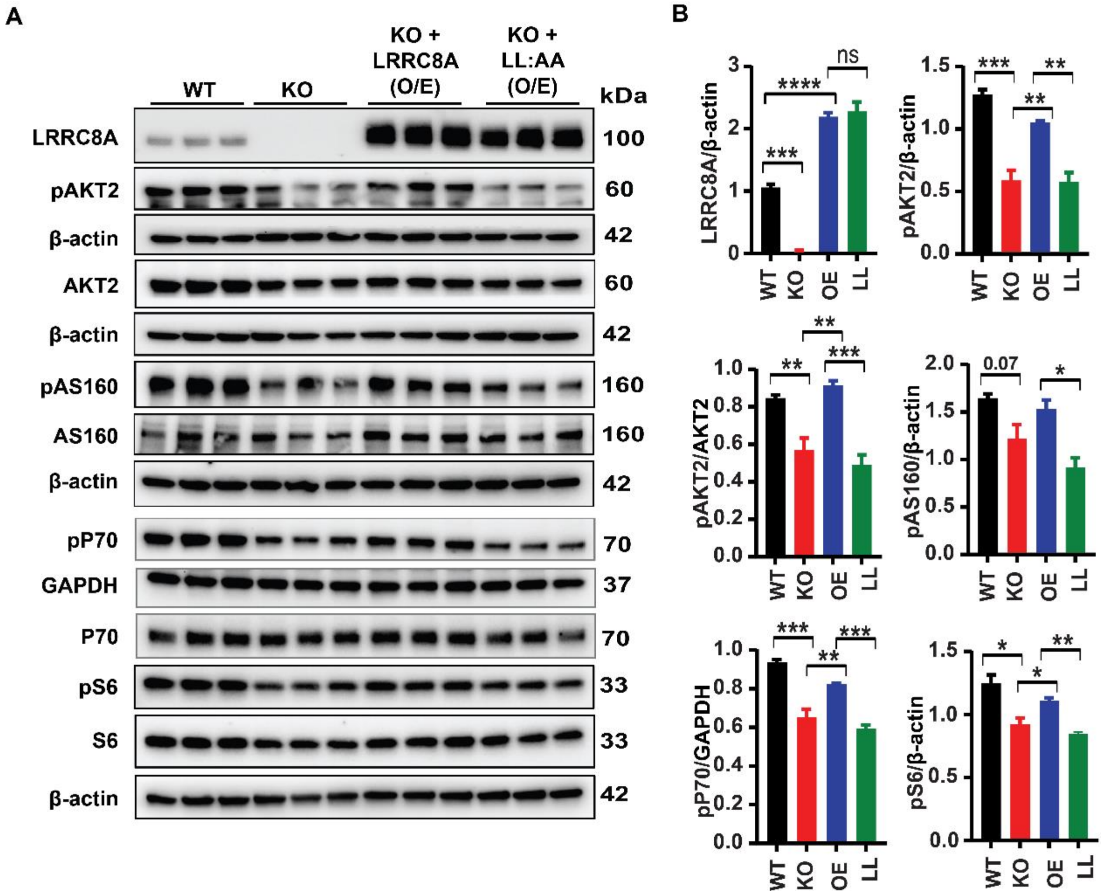

Figure S8. Re-expression of LRRC8A-LL:AA in LRRC8A null cells fail to restore PI3K-AKT-mTOR signaling.

A, Western blots of LRRC8A, pAKT2, AKT2, pAS160, AS160, pP70, P70, pS6, S6, β-actin and GAPDH in WT C2C12, LRRC8A KO C2C12, LRRC8A KO + LRRC8A-3xFlag, LRRC8A KO + LRRC8A-L706A;L707A-3xFlag (LL:AA) C2C12 myotubes at basal condition. **B,** Densitometry quantification of WB (A). Statistical significance between the indicated groups were calculated with one-way ANOVA, Tukey's multiple comparisons test. Error bars represent mean ± s.e.m. *, p<0.05, **, p<0.01, ***, p<0.001, ****, p<0.0001. n = 3, independent experiments.

figure S9

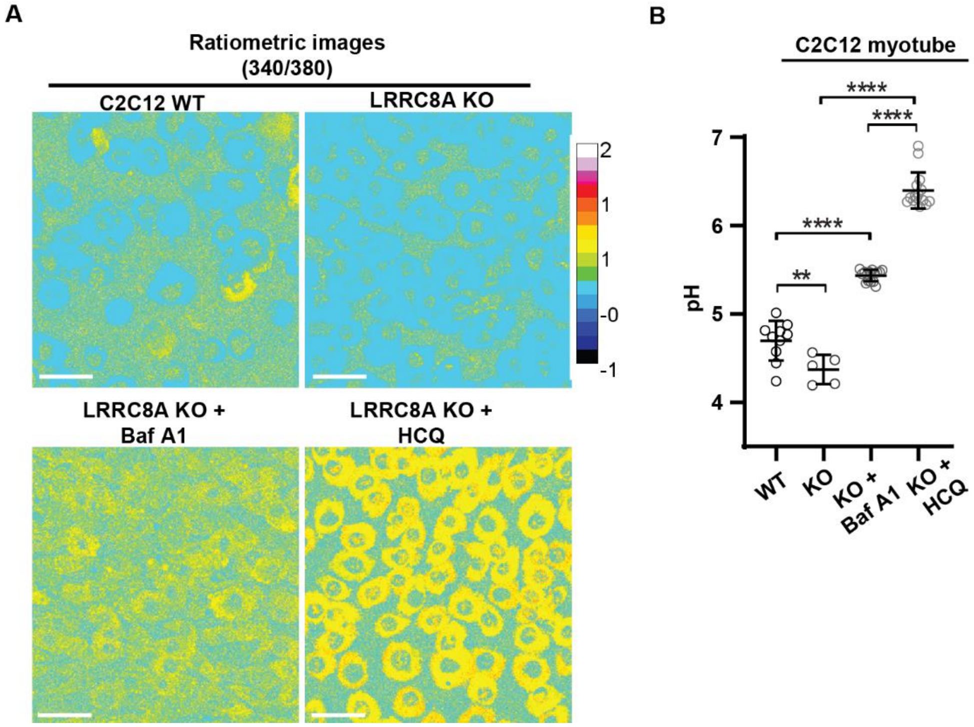

Figure S9. Lysosomal alkalinizing compounds increase lysosomal pH.

A, Ratiometric (Ex340/Ex380) fluorescence images of Lysosensor labeled images of WT C2C12 and LRRC8A KO myotubes after treated with or without HCQ (25 μM) or Baf A1 (10 nM) for 4 hours. Scale bar: 50 μm. **B,** Lysosomal pH of WT, LRRC8A KO, LRRC8A KO + BafA1 (10 nM) and LRRC8A KO + HCQ (25 μM) C2C12 myotubes which were determined from the non-linear least squares fit to the pH calibration curve. Scatter dot plot displays field of view quantification for 10 (WT- 2 dishes), 5 (KO- 1 dish), 15 (KO + Baf A1, 3 dishes) and 15 (KO + HCQ, 3 dishes), imaged from 1-3 dishes per condition. Statistical significance between the indicated values were calculated using one-way Anova, Tukey's multiple comparisons test. Error bars represent standard deviation (SD). *, $P < 0.05$, **, $P < 0.01$, ***, $P < 0.001$, ****, $P < 0.0001$.

Table S1: RNA sequencing data of LRRC8A-3xFlag-KI and LRRC8A-L706A; L707A knock-in (LL:AA KI) primary myotube.

Table S2: IPA canonical pathway analysis of LRRC8A-3xFlag-KI and LRRC8A-L706A; L707A knock-in (LL:AA KI) primary myotube.

Table S3: List of antibodies and materials used in this study. A list of all primary antibodies, secondary antibodies, chemicals, cell lines, mouse strains, recombinant DNA and plasmids, and software sources and information used in this study.

SUPPLEMENTARY MOVIE S1. Stimulated Emission Depletion (STED) super resolution z-stack imaging was captured, and composite 3D imaging was made by using ImgaeJ. Showing the localization of LRRC8A in the membrane and LAMP1 positive lysosomes (Green) in transiently expressed LRRC8A-ALFA (Red) in LRRC8A KO C2C12 myoblast cells.

**Antibodies**		
**Reagent / Item**	**Source**	**Information**
Anti-AKT1	Cell Signaling Technology	Cat #2938; RRID:AB_915788
Anti-AKT2	Cell Signaling Technology	Cat #3063; RRID: AB_2225186
Anti-GAPDH	Cell Signaling Technology	Cat #5174; RRID: AB_10622025
Anti-Cathepsin D	R and D Systems	Cat# AF1029, RRID:AB_2087094
Anti- pAS160	Cell Signaling Technology	Cat#4288s, RRID:AB_10545274
Anti- AS160	Cell Signaling Technology	Cat#2670s, RRID:AB_2199375
Anti- p70 S6 Kinase	Cell Signaling Technology	Cat#9202s, RRID:AB_331676
Anti- p-p70 S6 Kinase	Cell Signaling Technology	Cat#9205s, RRID:AB_330944

Anti- pS6 Ribosomal	Cell Signaling Technology	Cat#5364s, RRID:AB_10694233
Anti- S6 Ribosomal	Cell Signaling Technology	Cat#2317s, RRID:AB_2238583
Anti- flag	Sigma-Aldrich	Cat#F3165, RRID:AB_259529
Anti-LAMP1	DSHB	Cat# 1d4b-S lot#2019, Cat# 1d4b-C lot#2024, RRID:AB_2134500
Anti-LAMP2	DSHB	Cat# ABL-93, RRID:AB_2134767
Anti-SQSTM1 / p62	Abcam	Cat# ab56416, RRID:AB_945626
Anti-LC3B	Novus	Cat# NB100-2220, RRID:AB_10003146
Anti-pAKT1^{Ser473}	Cell Signaling Technology	Cat #9018; RRID:AB_2629283
Anti-pAKT2^{Ser474}	Cell Signaling Technology	Cat #8599; RRID: AB_2630347
Anti-β-actin	Cell Signaling Technology	Cat #8457; RRID: AB_10950489
Anti-Na,K-ATPase	Cell Signaling Technology	Cat #3010; RRID: AB_2060983
Anti-HA	BioLegend	Cat# 901501, RRID:AB_2565006
Pierce™ Anti-HA Magnetic Beads	Thermo Fisher Scientific	Cat# 88836, RRID:AB_2749815
Anti-ALFA-ATTO-643 conjugated	NanaoTag biotechnologies	Cat#N1502-At643-L, RRID:AB_3075983
Anti-LRRC8A	Sah Lab (54) (manufactured by Pacific Immunology)	N/A
Anti-LRRC8B	T.J. Jentsch	N/A
Anti-LRRC8C	Proteintech	Cat #21601-1-AP; RRID: AB_10733648
Anti-LRRC8D	T.J. Jentsch	N/A

Anti-LRRC8E	T.J. Jentsch	N/A
Goat anti-Rat IgG (H+L) Secondary Antibody, HRP	Thermo Fisher Scientific	Cat# 31470, RRID:AB_228356
Invitrogen Donkey anti-goat IgG (H+L)-Horseradish Peroxidase Conjugate	Thermo Fisher Scientific	Cat #A15999; RRID: AB_2534673
Goat anti-rabbit IgG (H+L)-Horseradish Peroxidase Conjugate	Bio-Rad	Cat #1706515; RRID: AB_11125142
**Chemicals & Kit**		
Accutase	Corning	Cat#MT25058CI
Amphotericin B	Sigma-Aldrich	Cat #A2942
Bafilomycin A1	Sigma-Aldrich	Cat #B1793
Bovine serum albumin	Fisher BioReagents	Cat #BP9706-100
bFGF	Gibco	Cat#13256029
Collagen Type II	Gibco	Cat#17101-015
Clarity™ Western ECL Substrate	Bio-Rad	Cat #1705060
cOmplete™ ULTRA Protease Inhibitor Cocktail	Roche Diagnostics	Cat #06538304001
DAPI	Invitrogen	Cat#D1306
Dispase	Roche	Cat#61206900
DC™ Protein Assay Kit I	Bio-Rad	Cat #5000111
DMEM/F12	Gibco	Cat#11330-032
DMEM	Gibco	Cat#11965
DNase digestion kit	Thermo Fisher Scientific	Cat#12185010
Nigericin	Sigma-Aldrich	Cat#N7143-5MG
HumulinR	Novolin	Cat#NDC-0169-1833-11
Insulin	Cell Applications	Cat#128-100
Insulin-Transferrin-Selenium (ITS -G)	Thermo Fisher Scientific	Cat#41400-045
4X Laemmli Sample Buffer	Bio-Rad	Cat #1610747

Lipofectamine 2000	Thermo Fisher Scientific	Cat#11668-019
LysoView 550	Biotium	Cat#70083
LysoSensor Yellow/Blue DND-160	Thermo Fisher Scientific	Cat#7545
Matrigel	Corning	Cat#356231
Penicillin / streptomycin	Thermo Fisher Scientific	Cat #15140122
PhosSTOP™	Roche Diagnostics	Cat #4906845001
RNA isolation (PureLink^{TM} RNA mini kit)	Invitrogen	Cat #12183018A
Tris/glycine buffer	Bio-Rad	Cat #1610771
Tris/glycine/SDS running buffer	Bio-Rad	Cat #1610772
µMACS™ and MultiMACS™ DYKDDDDK Isolation Kits	Miltenyi Biotech	Cat#130-101-591
**Cell Lines**		
C2C12 Cell line	ATCC	CRL-1772,<br>RRID:CVCL_0188
C2C12-LRRC8A KO	Sah Lab (20)	NA
HEK293	ATCC	CRL-1573, RRID:CVCL_0045
HEK-LRRC8A KO	Sah Lab (63)	N/A
HeLa quintuple KO	Zhaozhu Qiu, John Hopkins, Baltimore, MD, USA	N/A
**Experimental Models: Mouse/Strains**		
Mouse: C57BL/6N	Charles River Laboratories	Cat #027
Mouse: *Lrrc8a*$^{fl/fl}$ (*Swell1*$^{fl/fl}$)	Sah Lab (19) Washington University, Saint Louis	N/A
Mouse: LRRC8A-3xFlag-KI	Sah Lab, Washington University, Saint Louis	N/A
Mouse: LRRC8A-L706A;L707A-3xFlag-KI	Sah Lab, Washington University, Saint Louis	N/A

Mouse: LAMP1-RFP-1xFlag-TEV-HA	Diwan Lab, Washington University, Saint Louis	N/A
**Recombinant DNA and plasmids**		
Ad5-CMV-Cre-eGFP	University of Iowa viral vector core facility	N/A
Ad5-CMV-eGFP	University of Iowa viral vector core facility	N/A
Ad5-U6-scramble-mCherry	University of Iowa viral vector core facility	N/A
Ad5-mCherry-U6-shLRRC8A	University of Iowa viral vector core facility	N/A
Ad5-mPGK-mLrrc8a-(L706A)(L707A)-3xFlag	Vector Biolabs	N/A
Ad5-mPGK-mLrrc8a-3xFlag	Vector Biolabs	N/A
Ad(RGD)-CMV-iCre	Vector Biolabs	N/A
LRRC8C-P2A-mCherry	Vector Biolabs	N/A
pLV-CMV-rLAMP-RFP-PGK-puro	Diwan Lab, Washington University, Saint Louis	N/A
LRRC8A-ALFA-IRES-EGFP	Brohawn lab, University of California Berkeley	N/A
LRRC8A-ALFA	Brohawn lab, University of California Berkeley	N/A
LRRC8A-L706A;L707A-ALFA	VectorBuilder	N/A
**Software**		
BioRender	https://www.biorender.com/	**N/A**
Fiji	https://imagej.net/ – Schindelin *et al.* (64)	N/A
Prism	GraphPad Software	Version 10.2.0
pClamp	Molecular Devices	Version 10.4

table S3: List of antibodies and materials used in this study

<u>**Data supporting figures**</u>

Used for the quantification of Fig. 7

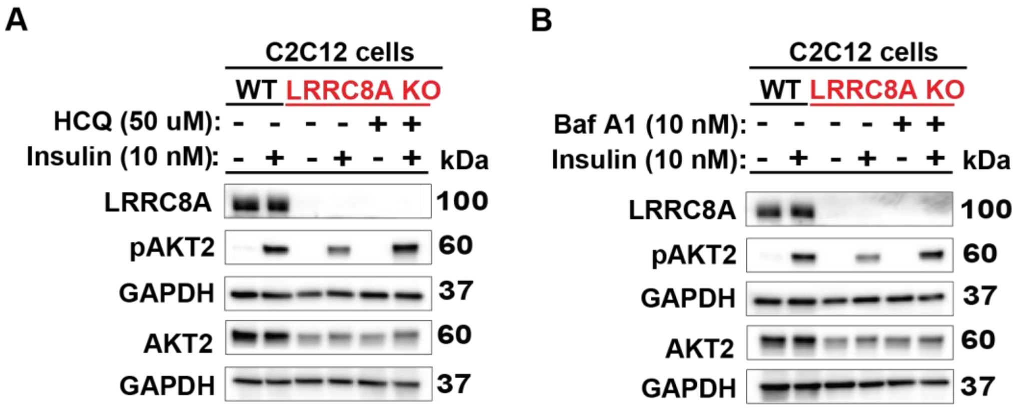

Used for the quantification of supplementary fig. 3

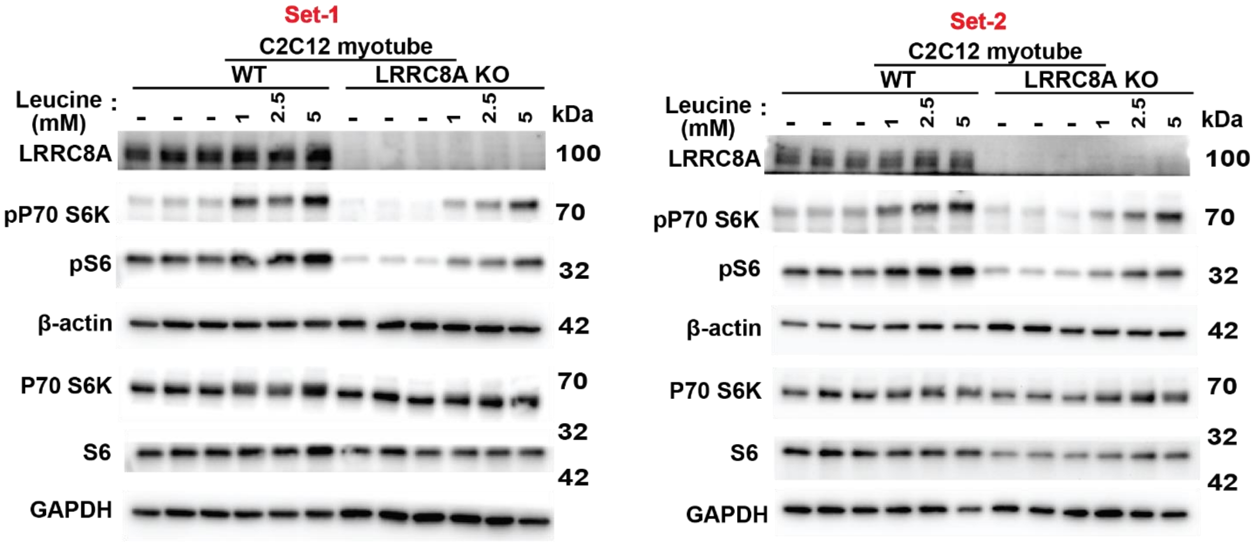

REFERENCES AND NOTES

1. L. Bar-Peled, L. D. Schweitzer, R. Zoncu, D. M. Sabatini, Ragulator is a GEF for the rag GTPases that signal amino acid levels to mTORC1. *Cell* **150**, 1196–1208 (2012).

2. C. Settembre, A. Fraldi, D. L. Medina, A. Ballabio, Signals from the lysosome: A control centre for cellular clearance and energy metabolism. *Nat. Rev. Mol. Cell Biol.* **14**, 283–296 (2013).

3. R. Zoncu, A. Efeyan, D. M. Sabatini, mTOR: From growth signal integration to cancer, diabetes and ageing. *Nat. Rev. Mol. Cell Biol.* **12**, 21–35 (2011).

4. E. L. Eskelinen, P. Saftig, Autophagy: A lysosomal degradation pathway with a central role in health and disease. *Biochim. Biophys. Acta* **1793**, 664–673 (2009).

5. T. O. Berg, M. Fengsrud, P. E. Strømhaug, T. Berg, P. O. Seglen, Isolation and characterization of rat liver amphisomes. Evidence for fusion of autophagosomes with both early and late endosomes. *J. Biol. Chem.* **273**, 21883–21892 (1998).

6. B. P. Lawrence, W. J. Brown, Autophagic vacuoles rapidly fuse with pre-existing lysosomes in cultured hepatocytes. *J. Cell Sci.* **102** (Pt. 3), 515–526 (1992).

7. A. A. Soyombo, S. Tjon-Kon-Sang, Y. Rbaibi, E. Bashllari, J. Bisceglia, S. Muallem, K. Kiselyov, TRP-ML1 regulates lysosomal pH and acidic lysosomal lipid hydrolytic activity. *J. Biol. Chem.* **281**, 7294–7301 (2006).

8. C. Curcio-Morelli, F. A. Charles, M. C. Micsenyi, Y. Cao, B. Venugopal, M. F. Browning, K. Dobrenis, S. L. Cotman, S. U. Walkley, S. A. Slaugenhaupt, Macroautophagy is defective in mucolipin-1-deficient mouse neurons. *Neurobiol. Dis.* **40**, 370–377 (2010).

9. M. Sun, E. Goldin, S. Stahl, J. L. Falardeau, J. C. Kennedy, J. S. Acierno Jr., C. Bove, C. R. Kaneski, J. Nagle, M. C. Bromley, M. Colman, R. Schiffmann, S. A. Slaugenhaupt, Mucolipidosis type IV is caused by mutations in a gene encoding a novel transient receptor potential channel. *Hum. Mol. Genet.* **9**, 2471–2478 (2000).

10. M. T. Bassi, M. Manzoni, E. Monti, M. T. Pizzo, A. Ballabio, G. Borsani, Cloning of the gene encoding a novel integral membrane protein, mucolipidin-and identification of the two major founder mutations causing mucolipidosis type IV. *Am. J. Hum. Genet.* **67**, 1110–1120 (2000).

11. S. Bose, C. de Heus, M. E. Kennedy, F. Wang, T. J. Jentsch, J. Klumperman, T. Stauber, Impaired autophagic clearance with a gain-of-function variant of the lysosomal Cl^-/H^+ exchanger ClC-7. *Biomolecules* **13**, 1799 (2023).

12. J. Osei-Owusu, J. Yang, K. H. Leung, Z. Ruan, W. Lü, Y. Krishnan, Z. Qiu, Proton-activated chloride channel PAC regulates endosomal acidification and transferrin receptor-mediated endocytosis. *Cell Rep.* **34**, 108683 (2021).

13. F. Ullrich, S. Blin, K. Lazarow, T. Daubitz, J. P. von Kries, T. J. Jentsch, Identification of TMEM206 proteins as pore of PAORAC/ASOR acid-sensitive chloride channels. *eLife* **8**, e49187 (2019).

14. M. Hu, P. Li, C. Wang, X. Feng, Q. Geng, W. Chen, M. Marthi, W. Zhang, C. Gao, W. Reid, J. Swanson, W. Du, R. I. Hume, H. Xu, Parkinson's disease-risk protein TMEM175 is a proton-activated proton channel in lysosomes. *Cell* **185**, 2292–2308.e20 (2022).

15. F. K. Voss, F. Ullrich, J. Münch, K. Lazarow, D. Lutter, N. Mah, M. A. Andrade-Navarro, J. P. von Kries, T. Stauber, T. J. Jentsch, Identification of LRRC8 heteromers as an essential component of the volume-regulated anion channel VRAC. *Science* **344**, 634–638 (2014).

16. Z. Qiu, A. E. Dubin, J. Mathur, B. Tu, K. Reddy, L. J. Miraglia, J. Reinhardt, A. P. Orth, A. Patapoutian, SWELL1, a plasma membrane protein, is an essential component of volume-regulated anion channel. *Cell* **157**, 447–458 (2014).

17. P. Li, M. Hu, C. Wang, X. Feng, Z. Zhao, Y. Yang, N. Sahoo, M. Gu, Y. Yang, S. Xiao, R. Sah, T. L. Cover, J. Chou, R. Geha, F. Benavides, R. I. Hume, H. Xu, LRRC8 family proteins within lysosomes regulate cellular osmoregulation and enhance cell survival to multiple physiological stresses. *Proc. Natl. Acad. Sci. U.S.A.* **117**, 29155–29165 (2020).

18. G. M. Lenk, Y. N. Park, R. Lemons, E. Flynn, M. Plank, C. M. Frei, M. J. Davis, B. Gregorka, J. A. Swanson, M. H. Meisler, J. O. Kitzman, CRISPR knockout screen implicates three genes in lysosome function. *Sci. Rep.* **9**, 9609 (2019).

19. Y. Zhang, L. Xie, S. K. Gunasekar, D. Tong, A. Mishra, W. J. Gibson, C. Wang, T. Fidler, B. Marthaler, A. Klingelhutz, E. D. Abel, I. Samuel, J. K. Smith, L. Cao, R. Sah, SWELL1 is a regulator of adipocyte size, insulin signalling and glucose homeostasis. *Nat. Cell Biol.* **19**, 504–517 (2017).

20. A. Kumar, L. Xie, C. M. Ta, A. O. Hinton, S. K. Gunasekar, R. A. Minerath, K. Shen, J. M. Maurer, C. E. Grueter, E. D. Abel, G. Meyer, R. Sah, SWELL1 regulates skeletal muscle cell size, intracellular signaling, adiposity and glucose metabolism. *eLife* **9**, e58941 (2020).

21. L. Chen, T. M. Becker, U. Koch, T. Stauber, The LRRC8/VRAC anion channel facilitates myogenic differentiation of murine myoblasts by promoting membrane hyperpolarization. *J. Biol. Chem.* **294**, 14279–14288 (2019).

22. K. I. López-Cayuqueo, R. Planells-Cases, M. Pietzke, A. Oliveras, S. Kempa, S. Bachmann, T. J. Jentsch, Renal deletion of LRRC8/VRAC channels induces proximal tubulopathy. *J. Am. Soc. Nephrol.* **33**, 1528–1545 (2022).

23. H. Götzke, M. Kilisch, M. Martínez-Carranza, S. Sograte-Idrissi, A. Rajavel, T. Schlichthaerle, N. Engels, R. Jungmann, P. Stenmark, F. Opazo, S. Frey, The ALFA-tag is a highly versatile tool for nanobody-based bioscience applications. *Nat. Commun.* **10**, 4403 (2019).

24. S. Zhang, X. Zeng, M. Ren, X. Mao, S. Qiao, Novel metabolic and physiological functions of branched chain amino acids: A review. *J. Anim. Sci. Biotechnol.* **8**, 10 (2017).

25. J. Dyachok, S. Earnest, E. N. Iturraran, M. H. Cobb, E. M. Ross, Amino acids regulate mTORC1 by an obligate two-step mechanism. *J. Biol. Chem.* **291**, 22414–22426 (2016).

26. L. Deldicque, C. Sanchez Canedo, S. Horman, I. De Potter, L. Bertrand, L. Hue, M. Francaux, Antagonistic effects of leucine and glutamine on the mTOR pathway in myogenic C2C12 cells. *Amino Acids* **35**, 147–155 (2008).

27. A. F. Alghanem, J. Abello, J. M. Maurer, A. Kumar, C. M. Ta, S. K. Gunasekar, U. Fatima, C. Kang, L. Xie, O. Adeola, M. Riker, M. Elliot-Hudson, R. A. Minerath, C. E. Grueter, R. F. Mullins, A. N. Stratman, R. Sah, The SWELL1-LRRC8 complex regulates endothelial AKT-eNOS signaling and vascular function. *eLife* **10**, e61313 (2021).

28. S. Vergarajauregui, R. Puertollano, Two di-leucine motifs regulate trafficking of mucolipin-1 to lysosomes. *Traffic* **7**, 337–353 (2006).

29. E. Cerniauskas, M. Kurzawa-Akanbi, L. Xie, D. Hallam, M. Moya-Molina, K. White, D. Steel, M. Doherty, P. Whitfield, J. Al-Aama, L. Armstrong, D. Kavanagh, J. D. Lambris, V. I. Korolchuk, C. Harris, M. Lako, Complement modulation reverses pathology in Y402H-retinal pigment epithelium cell model of age-related macular degeneration by restoring lysosomal function. *Stem Cells Transl. Med.* **9**, 1585–1603 (2020).

30. M. K. Pandey, Exploring pro-inflammatory immunological mediators: Unraveling the mechanisms of neuroinflammation in lysosomal storage diseases. *Biomedicine* **11**, 1067 (2023).

31. W. J. Liu, Z.-h. Li, X.-c. Chen, X.-l. Zhao, Z. Zhong, C. Yang, H.-l. Wu, N. An, W.-y. Li, H.-f. Liu, Blockage of the lysosome-dependent autophagic pathway contributes to complement membrane attack complex-induced podocyte injury in idiopathic membranous nephropathy. *Sci. Rep.* **7**, 8643 (2017).

32. B. C. King, K. Kulak, U. Krus, R. Rosberg, E. Golec, K. Wozniak, M. F. Gomez, E. Zhang, D. J. O'Connell, E. Renström, A. M. Blom, Complement component C3 is highly expressed in human pancreatic islets and prevents β cell death via ATG16L1 interaction and autophagy regulation. *Cell Metab.* **29**, 202–210.e6 (2019).

33. M. Jung, J. Lee, H. Y. Seo, J. S. Lim, E. K. Kim, Cathepsin inhibition-induced lysosomal dysfunction enhances pancreatic beta-cell apoptosis in high glucose. *PLOS ONE* **10**, e0116972 (2015).

34. E. C. Coffey, M. Astumian, S. S. Alrowaished, C. Schaffer, C. A. Henry, Lysosomal function impacts the skeletal muscle extracellular matrix. *J. Dev. Biol.* **9**, 52 (2021).

35. R. A. Haeusler, T. E. McGraw, D. Accili, Biochemical and cellular properties of insulin receptor signalling. *Nat. Rev. Mol. Cell Biol.* **19**, 31–44 (2018).

36. B. Desbuquois, S. López, M. Janicot, H. Burlet, B. de Gallé, F. Fouque, Role of acidic subcellular compartments in the degradation of internalized insulin and in the recycling of the internalized insulin receptor in liver cells: In vivo and in vitro studies. *Diabete Metab.* **18**, 104–112 (1992).

37. F. G. S. Toledo, R. G. Miller, N. L. Helbling, Y. Zhang, J. P. DeLany, The effects of hydroxychloroquine on insulin sensitivity, insulin clearance and inflammation in insulin-resistant adults: A randomized trial. *Diabetes Obes. Metab.* **23**, 1252–1261 (2021).

38. A. El-Solia, K. Al-Otaibi, A. K. Ai-Hwiesh, Hydroxychloroquine-induced hypoglycaemia in non-diabetic renal patient on peritoneal dialysis. *BMJ Case Rep.* **2018**, bcr-2017-223639 (2018).

39. D. U. Cansu, C. Korkmaz, Hypoglycaemia induced by hydroxychloroquine in a non-diabetic patient treated for RA. *Rheumatology* **47**, 378–379 (2008).

40. A. P. Bevan, A. Krook, J. Tikerpae, P. J. Seabright, K. Siddle, G. D. Smith, Chloroquine extends the lifetime of the activated insulin receptor complex in endosomes. *J. Biol. Chem.* **272**, 26833–26840 (1997).

41. A. P. Bevan, J. R. Christensen, J. Tikerpae, G. D. Smith, Chloroquine augments the binding of insulin to its receptor. *Biochem. J.* **311** (Pt. 3), 787–795 (1995).

42. D. J. Colacurcio, R. A. Nixon, Disorders of lysosomal acidification—The emerging role of v-ATPase in aging and neurodegenerative disease. *Ageing Res. Rev.* **32**, 75–88 (2016).

43. J. Zhang, W. Zeng, Y. Han, W. R. Lee, J. Liou, Y. Jiang, Lysosomal LAMP proteins regulate lysosomal pH by direct inhibition of the TMEM175 channel. *Mol. Cell* **83**, 2524–2539.e7 (2023).

44. X. Leray, J. K. Hilton, K. Nwangwu, A. Becerril, V. Mikusevic, G. Fitzgerald, A. Amin, M. R. Weston, J. A. Mindell, Tonic inhibition of the chloride/proton antiporter ClC-7 by PI(3,5)P2 is crucial for lysosomal pH maintenance. *eLife* **11**, e74136 (2022).

45. A. R. Graves, P. K. Curran, C. L. Smith, J. A. Mindell, The Cl^-/H^+ antiporter ClC-7 is the primary chloride permeation pathway in lysosomes. *Nature* **453**, 788–792 (2008).

46. E. R. Nicoli, M. R. Weston, M. Hackbarth, A. Becerril, A. Larson, W. M. Zein, P. R. Baker II, J. D. Burke, H. Dorward, M. Davids, Y. Huang, D. R. Adams, P. M. Zerfas, D. Chen, T. C. Markello, C. Toro, T. Wood, G. Elliott, M. Vu, W. Zheng, L. J. Garrett, C. J. Tifft, W. A. Gahl, D. L. Day-Salvatore, J. A. Mindell, M. C. V. Malicdan, M. T. Acosta, D. R. Adams, P. Agrawal, M. E. Alejandro, P. Allard, J. Alvey, A. Andrews, E. A. Ashley, M. S. Azamian, C. A. Bacino, G. Bademci, E. Baker, A. Balasubramanyam, D. Baldridge, J. Bale, D. Barbouth, G. F. Batzli, P. Bayrak-Toydemir, A. H. Beggs, G. Bejerano, H. J. Bellen, J. A. Bernstein, G. T. Berry, A. Bican, D. P. Bick, C. L. Birch, S. Bivona, J. Bohnsack, C. Bonnenmann, D. Bonner, B. E. Boone, B. L. Bostwick, L. Botto, L. C. Briere, E. Brokamp, D. M. Brown, M. Brush, E. A. Burke, L. C. Burrage, M. J. Butte, J. Carey, O. Carrasquillo, T. C. P. Chang, H. T. Chao, G. D. Clark, T. R. Coakley, L. A. Cobban, J. D. Cogan, F. S. Cole, H. A. Colley, C. M. Cooper, H. Cope, W. J. Craigen, P. D'Souza, S. Dasari, M. Davids, J. G. Dayal, E. C. Dell'Angelica, S. U. Dhar, N. Dorrani, D. C. Dorset, E. D. Douine, D. D. Draper, L. Duncan, D. J. Eckstein, L. T. Emrick, C. M. Eng, C. Esteves, T. Estwick, L. Fernandez, C. Ferreira, E. L. Fieg, P. G. Fisher, B. L. Fogel, I. Forghani, L. Fresard, W. A. Gahl, R. A. Godfrey, A. M. Goldman, D. B. Goldstein, J. P. F. Gourdine, A. Grajewski, C. A. Groden, A. L. Gropman, M. Haendel, R. Hamid, N. A. Hanchard, N. Hayes, F. High, I. A. Holm, J. Hom, A. Huang, Y. Huang, R. Isasi, F. Jamal, Y. H. Jiang, J. M. Johnston, A. L. Jones, L. Karaviti, E. G. Kelley, D. Kiley, D. M. Koeller, I. S. Kohane, J. N. Kohler, D. Krakow, D. M. Krasnewich, S. Korrick, M. Koziura, J. B. Krier, J. E. Kyle, S. R. Lalani, B. Lam, B. C. Lanpher, I. R. Lanza, C. C. Lau, J. Lazar, K. LeBlanc, B. H. Lee, H. Lee, R. Levitt, S. E. Levy, R. A. Lewis, S. A. Lincoln, P. Liu, X. Z. Liu, N. Longo, S. K. Loo, J. Loscalzo, R. L. Maas, E. F. Macnamara,

C. A. MacRae, V. V. Maduro, M. M. Majcherska, M. C. V. Malicdan, L. A. Mamounas, T. A. Manolio, R. Mao, T. C. Markello, R. Marom, G. Marth, B. A. Martin, M. G. Martin, J. A. Martínez-Agosto, S. Marwaha, T. May, J. McCauley, A. McConkie-Rosell, C. E. McCormack, A. T. McCray, T. O. Metz, M. Might, E. Morava-Kozicz, P. M. Moretti, M. Morimoto, J. J. Mulvihill, D. R. Murdock, A. Nath, S. F. Nelson, J. S. Newberry, J. H. Newman, S. K. Nicholas, D. Novacic, D. Oglesbee, J. P. Orengo, L. Pace, S. Pak, J. C. Pallais, C. G. S. Palmer, J. C. Papp, N. H. Parker, J. A. Phillips III, J. E. Posey, J. H. Postlethwait, L. Potocki, B. N. Pusey, A. Quinlan, A. N. Raja, G. Renteria, C. M. Reuter, L. Rives, A. K. Robertson, L. H. Rodan, J. A. Rosenfeld, R. K. Rowley, M. Ruzhnikov, R. Sacco, J. B. Sampson, S. L. Samson, M. Saporta, J. Schaechter, T. Schedl, K. Schoch, D. A. Scott, L. Shakachite, P. Sharma, V. Shashi, K. Shields, J. Shin, R. Signer, C. H. Sillari, E. K. Silverman, J. S. Sinsheimer, K. Sisco, K. S. Smith, L. Solnica-Krezel, R. C. Spillmann, J. M. Stoler, N. Stong, J. A. Sullivan, S. Sutton, D. A. Sweetser, H. K. Tabor, C. P. Tamburro, Q. K. G. Tan, M. Tekin, F. Telischi, W. Thorson, C. J. Tifft, C. Toro, A. A. Tran, T. K. Urv, M. Velinder, D. Viskochil, T. P. Vogel, C. E. Wahl, N. M. Walley, C. A. Walsh, M. Walker, J. Wambach, J. Wan, L. K. Wang, M. F. Wangler, P. A. Ward, K. M. Waters, B. J. M. Webb-Robertson, D. Wegner, M. Westerfield, M. T. Wheeler, A. L. Wise, L. A. Wolfe, J. D. Woods, E. A. Worthey, S. Yamamoto, J. Yang, A. J. Yoon, G. Yu, D. B. Zastrow, C. Zhao, S. Zuchner, Lysosomal storage and albinism due to effects of a de novo CLCN7 variant on lysosomal acidification. *Am. J. Hum. Genet.* **104**, 1127–1138 (2019).

47. Y. Mizunoe, M. Kobayashi, R. Tagawa, Y. Nakagawa, H. Shimano, Y. Higami, Association between lysosomal dysfunction and obesity-related pathology: A key knowledge to prevent metabolic syndrome. *Int. J. Mol. Sci.* **20**, 3688 (2019).

48. K. R. Menikdiwela, L. Ramalingam, F. Rasha, S. Wang, J. M. Dufour, N. S. Kalupahana, K. K. S. Sunahara, J. O. Martins, N. Moustaid-Moussa, Autophagy in metabolic syndrome: Breaking the wheel by targeting the renin-angiotensin system. *Cell Death Dis.* **11**, 87 (2020).

49. L. Y. Ma, S. F. Liu, Y. G. Guo, Z. Q. Ma, Y. Li, S. J. Wang, Y. Niu, M. Li, J. J. Zhai, S. H. Shang, Y. L. Lv, Q. M. Qu, Diabetes influences the fusion of autophagosomes with lysosomes in SH-SY5Y cells and induces Aβ deposition and cognitive dysfunction in STZ-induced diabetic rats. *Behav. Brain Res.* **442**, 114286 (2023).

50. S. Marshall, J. M. Olefsky, Effects of lysosomotropic agents on insulin interactions with adipocytes. Evidence for a lysosomal pathway for insulin processing and degradation. *J. Biol. Chem.* **254**, 10153–10160 (1979).

51. S. Marshall, D. A. Podlecki, J. M. Olefsky, Low pH accelerates dissociation of receptor-bound insulin. *Endocrinology* **113**, 37–42 (1983).

52. P. Rachubik, D. Rogacka, I. Audzeyenka, M. Typiak, M. Wysocka, M. Szrejder, A. Lesner, A. Piwkowska, Role of lysosomes in insulin signaling and glucose uptake in cultured rat podocytes. *Biochem. Biophys. Res. Commun.* **679**, 145–159 (2023).

53. Z. Guo, C. Valenzuela Ripoll, A. Picataggi, D. R. Rawnsley, M. Ozcan, J. A. Chirinos, E. Chendamarai, A. Girardi, T. Riehl, H. Evie, A. Diab, A. Kovacs, K. Hyrc, X. Ma, A. Asnani, S. V. Shewale, M. Scherrer-Crosbie, L. A. Cowart, J. S. Parks, L. Zhao, D. Gordon, F. Ramirez-Valle, K. B. Margulies, T. P. Cappola, A. A. Desai, L. N. Pedersen, C. Bergom, N. O. Stitziel, M. P. Rettig, J. F. DiPersio, S. Hajny, C. Christoffersen, A. Diwan, A. Javaheri, Apolipoprotein M attenuates anthracycline cardiotoxicity and lysosomal injury. *JACC Basic Transl. Sci.* **8**, 340–355 (2023).

54. C. Kang, L. Xie, S. K. Gunasekar, A. Mishra, Y. Zhang, S. Pai, Y. Gao, A. Kumar, A. W. Norris, S. B. Stephens, R. Sah, SWELL1 is a glucose sensor regulating β-cell excitability and systemic glycaemia. *Nat. Commun.* **9**, 367 (2018).

55. X. Zhang, K. Garbett, K. Veeraraghavalu, B. Wilburn, R. Gilmore, K. Mirnics, S. S. Sisodia, A role for presenilins in autophagy revisited: Normal acidification of lysosomes in cells lacking PSEN1 and PSEN2. *J. Neurosci.* **32**, 8633–8648 (2012).

56. J. Canton, S. Grinstein, Measuring lysosomal pH by fluorescence microscopy. *Methods Cell Biol.* **126**, 85–99 (2015).

57. K. Neikirk, Z. Vue, P. Katti, B. I. Rodriguez, S. Omer, J. Shao, T. Christensen, E. Garza Lopez, A. Marshall, C. B. Palavicino-Maggio, J. Ponce, A. F. Alghanem, L. Vang, T. Barongan, H. K. Beasley, T. Rodman, D. Stephens, M. Mungai, M. Correia, V. Exil, S. Damo, S. A. Murray, A. Crabtree, B. Glancy, R. O. Pereira, E. D. Abel, A. O. Hinton Jr.,

Systematic transmission electron microscopy-based identification and 3D reconstruction of cellular degradation machinery. *Adv. Biol.* **7**, e2200221 (2023).

58. J. Lam, P. Katti, M. Biete, M. Mungai, S. AshShareef, K. Neikirk, E. Garza Lopez, Z. Vue, T. A. Christensen, H. K. Beasley, T. A. Rodman, S. A. Murray, J. L. Salisbury, B. Glancy, J. Shao, R. O. Pereira, E. D. Abel, A. Hinton Jr., A universal approach to analyzing transmission electron microscopy with ImageJ. *Cells* **10**, 2177 (2021).

59. M. Abu-Remaileh, G. A. Wyant, C. Kim, N. N. Laqtom, M. Abbasi, S. H. Chan, E. Freinkman, D. M. Sabatini, Lysosomal metabolomics reveals V-ATPase- and mTOR-dependent regulation of amino acid efflux from lysosomes. *Science* **358**, 807–813 (2017).

60. S. K. Gunasekar, L. Xie, A. Kumar, J. Hong, P. R. Chheda, C. Kang, D. M. Kern, C. My-Ta, J. Maurer, J. Heebink, E. E. Gerber, W. J. Grzesik, M. Elliot-Hudson, Y. Zhang, P. Key, C. A. Kulkarni, J. W. Beals, G. I. Smith, I. Samuel, J. K. Smith, P. Nau, Y. Imai, R. D. Sheldon, E. B. Taylor, D. J. Lerner, A. W. Norris, S. Klein, S. G. Brohawn, R. Kerns, R. Sah, Small molecule SWELL1 complex induction improves glycemic control and nonalcoholic fatty liver disease in murine type 2 diabetes. *Nat. Commun.* **13**, 784 (2022).

61. E. P. Homan, B. B. Brandão, S. Softic, A. El Ouaamari, B. T. O'Neill, R. N. Kulkarni, J. K. Kim, C. R. Kahn, Differential roles of FOXO transcription factors on insulin action in brown and white adipose tissue. *J. Clin. Invest.* **131**, e143328 (2021).

62. R. Steele, J. S. Wall, R. C. De Bodo, N. Altszuler, Measurement of size and turnover rate of body glucose pool by the isotope dilution method. *Am. J. Physiol.* **187**, 15–24 (1956).

63. J. Schindelin, I. Arganda-Carreras, E. Frise, V. Kaynig, M. Longair, T. Pietzsch, S. Preibisch, C. Rueden, S. Saalfeld, B. Schmid, J.-Y. Tinevez, D. J. White, V. Hartenstein, K. Eliceiri, P. Tomancak, A. Cardona, Fiji: An open-source platform for biological-image analysis. *Nat. Methods* **9**, 676–682 (2012).

64. R. Wang, Y. Lu, S. Gunasekar, Y. Zhang, C. J. Benson, M. W. Chapleau, R. Sah, F. M. Abboud, The volume-regulated anion channel (LRRC8) in nodose neurons is sensitive to acidic pH. *JCI Insight* **2**, e90632 (2017).
